# An Investigation of Lower Limb Representations Underlying Vision, Touch, and Proprioception in Body Integrity Identity Disorder

**DOI:** 10.3389/fpsyt.2020.00015

**Published:** 2020-02-25

**Authors:** Kayla D. Stone, Clara A. E. Kornblad, Manja M. Engel, H. Chris Dijkerman, Rianne M. Blom, Anouk Keizer

**Affiliations:** ^1^ Department of Experimental Psychology, Helmholtz Institute, Utrecht University, Utrecht, Netherlands; ^2^ Department of Psychiatry, Amsterdam UMC, Vrije Universiteit Amsterdam, Amsterdam, Netherlands

**Keywords:** xenomelia, body integrity dysphoria, lower limbs, body perception, multisensory, body ownership

## Abstract

Individuals with Body Integrity Identity Disorder (BIID) have a (non-psychotic) longstanding desire to amputate or paralyze one or more fully-functioning limbs, often the legs. This desire presumably arises from experiencing a mismatch between one’s perceived mental image of the body and the physical structural and/or functional boundaries of the body itself. While neuroimaging studies suggest a disturbed body representation network in individuals with BIID, few behavioral studies have looked at the manifestation of this disrupted lower limb representations in this population. Specifically, people with BIID feel like they are overcomplete in their current body. Perhaps sensory input, processed normally on and about the limb, cannot communicate with a higher-order model of the leg in the brain (which might be underdeveloped). We asked individuals who desire paralysis or amputation of the lower legs (and a group of age- and sex-matched controls) to make explicit and implicit judgments about the size and shape of their legs while relying on vision, touch, and proprioception. We hypothesized that BIID participants would mis-estimate the size of their affected leg(s) more than the same leg of controls. Using a multiple single-case analysis, we found no global differences in lower limb representations between BIID participants and controls. Thus, while people with BIID feel that part of the body is foreign, they can still make normal sensory-guided implicit and explicit judgments about the limb. Moreover, these results suggest that BIID is not a body image disorder, per se, and that an examination of leg representation does not uncover the disturbed bodily experience that individuals with BIID have.

## Introduction

Body Integrity Identity Disorder (BIID) is a rare, non-psychotic condition characterized by a persistent and strong desire to acquire a physical disability (1). This can include the (lesser known) variant which involves the desire for deafness (2) or blindness (3), but most reported cases of BIID involve the desire to amputate or paralyze one or more healthy limbs [e.g. (2–12)]. People with this variant of BIID (who will be the focus of the current report) presumably experience a mismatch between their internal mental image of the body and the external physical and functional boundaries of the body itself (4, 13). The condition is not a product of any apparent brain damage, most often manifests before adolescence, is more prevalent in males than females, and is reported more often for the lower limbs than for the upper limbs [see (14–17) for reviews]. While these individuals have normal sensory feedback (like vision, touch, and proprioception) from and about the affected limb (5, 11), they feel overcomplete with that limb, and that it is redundant in the bodily experience. Instead, they feel that removing the limb [or at least removing the functional abilities of the limb(s)] will make them feel complete, such that the external physical body would then match their, presumably innate, internal image of how the body should be. In other words, people with BIID likely experience an incongruence between their biological body and internal body representation.

Therefore, is BIID a product of a disturbed internal body representation? Neuroimaging evidence suggests that this may be the case. Specifically, individuals with amputation-variant BIID [i.e. those who desire amputation of a limb, also known as xenomelia (10)] have structural and functional alterations (compared to healthy control participants) in the body-representation network, specific to the superior parietal lobule (SPL), primary somatosensory cortex (SI), secondary somatosensory cortex (SII), supplementary motor area, and the paracentral lobule (which contains the SI leg representation), premotor cortex (PMC), and insula, and also other subcortical areas, perhaps more involved in sensorimotor control, like the cerebellum, putamen, caudate nucleus, pallidum, thalamus, and basal ganglia (11, 18–21). In addition, people with BIID show a reduction in activity of the SPL (10) and PMC (20) when being touched on the affected compared to the unaffected leg and to the legs of control participants. These brain areas are critical for integrating sensory input and maintaining models of the body, specifically for feeling ownership over a body part (22–25). So, although they can *feel* tactile input on their legs, there seems to be some fault at registering that information with a higher-level leg representation in the brain. Recently, Oddo-Sommerfeld and colleagues (12) found differential brain activity specific to most of the aforementioned body representation regions when individuals with BIID viewed images of themselves modified to look like a lower-limb amputee (desired body type) compared to controls viewing the same modified image of themselves (non-desired body type). Specifically, brain activity accurately predicted group membership, i.e. whether the participant belonged to the BIID group or control group. In addition, they found (unexpected) differential activation of lower- and higher- visual areas in the occipital lobe too. These studies indeed reveal an underlying disturbed body-representation network that could manifest as (or contribute to) an incongruence between the perceived internal representation of the body and the actual body. In line with this, many BIID researchers suggest that BIID might be indicative of “an inability of the brain to “map” or integrate sensation from the limb into [higher-order] body maps” (26) on page 3, (10, 20, 21, 27). Specifically, these higher-order body maps underlying visual and sensorimotor (like somatosensory, proprioceptive) information might be disrupted, such that they are incomplete or underdeveloped, in BIID.

Yet, few behavioral studies have investigated the manifestation of this disordered body representation in BIID individuals. We know that, at least for the amputation-variant, these individuals have an implicit preference for amputated versus intact bodies (7), have a more vivid rubber foot illusion for the affected foot (8), show impaired temporal-spatial processing of tactile stimuli for the affected leg (5), have an increased skin conductance response (SCR) to stimuli contacting the affected (but not the unaffected) limb (9, 28), and a reduced SCR response to stimuli *approaching* the affected limb (28). These studies, in conjunction with the neuroimaging results, do suggest that the origins of BIID might be a consequence of disturbed integration of bottom-up information (like vision, touch, proprioception) with top-down information, like higher order model(s) of the affected body part. What remains unknown, however, is how these models underlying sensory input for the affected body parts look in BIID. Is BIID a body representation disorder (specifically, is the perception of the affected leg distorted)? And if it is a problem with primary sensory input reaching the higher order representation(s), at which source does it start to falter? The one that underlies vision, or touch, or proprioception, if any? This urges one to investigate leg representations underlying these sensory modalities in BIID. We wanted to investigate this not only in those desiring amputation of their leg(s), but also in those with the paralysis-variant of BIID, since no studies, to our knowledge, have yet investigated body representations in people with paralysis-variant BIID.

One way to tap into the representation of the body is by asking people to make judgments about the size and shape of their body parts. Several investigations have revealed that healthy people have a distorted representation of their bodies, and this is dependent (at least partly) on the most reliable and dominant source of sensory information available when making judgments about that body part. People consistently overestimate the width of their hands when asked to make more implicit judgments about the body, like to localize points on their unseen hand (proprioceptive feedback) and underestimate tactile distances applied lengthwise on the hand (tactile feedback) but are accurate when asked to explicitly judge the shape of the hand when looking at pictures of it [visual feedback, i.e. the more conscious body image (29)]. The metric body representation of the lower limbs, however, has been much less studied (30–33). Though recently, we investigated the role that vision, touch, and proprioception play in making estimates about the underlying body representation of the lower limbs (33). Healthy participants made judgments about the size, shape, and location of landmarks on the legs while relying on different sensory input, and results revealed that body representations of the leg are also distorted. When asked to localize points on the leg while relying on its unseen position in space, participants perceived the upper legs to be longer/thinner than they are, but the lower legs to be squatter/shorter than they are. Distortions also ensued when asked to judge unseen tactile distances on the legs: tactile distances applied length-wise were underestimated more than those applied width-wise. Furthermore, when presented with different images of their legs onscreen, participants slightly overestimated their widths (length estimates were not tested). Thus, the underlying leg representations, underlying different weights of sensory input, show systematic distortions in healthy people.

Specifically, it has been proposed that perceiving the distance between two (tactile) points applied to a body part or localizing an unseen landmark on the body requires reference to a higher-order representation, i.e. the body model (34, 35). That is, the raw tactile and proprioceptive afferent information (e.g. about joint angle or skin stretch) is not informative about the size of the body part per se, so in order to estimate these tactile distances or localize one’s position in space, the brain needs to refer to and integrate with a higher-order representation of the size and shape of the body (34–36). We suspect that these higher-order representations (or the connections to them) could be underdeveloped in BIID. On the other hand, however, visual estimates about perceived body size, specifically when judging (distorted) pictures of the body, tend to be veridical in healthy individuals [e.g. (29, 37)], suggesting a distinction between (more implicit) somatosensory and (more explicit) visual representations of the body (29, 36, 38). In clinical conditions, estimates of body part size tend to mimic one’s internal and external experience of the body. For example, individuals with anorexia nervosa overestimate the distance between two tactile points on the body (39–41) but also overestimate body size when making more explicit judgments about images of their own body [e.g. 38,39; see (42) for review]. Similarly, individuals with complex regional pain syndrome (CRPS) overestimate body size in a template-matching task (43), reflecting their feeling that the limb feels bigger than it is. Yet, stroke patients who are asked to complete more implicit judgments about the affected body part, like localize the midpoint of their unseen arm, tend to underestimate its length (44). Individuals with BIID feel “overcomplete” on the outside, but like an amputee or paraplegic individual on the inside. Therefore, one might wonder whether these aforementioned tasks can shed lighten the hypothesized disturbances in body representations in BIID.

Therefore, in the current study, we explored leg representations underlying somatosensory and visual information about the body in a group of people with amputation- and paralysis-variant BIID. We employed the same tasks as used in Stone et al. (33). We hypothesized that participants with BIID would:

1. overestimated their disowned/affected leg(s) more than the/affected leg(s) more than the same leg(s) of controls when making conscious, visually-guided estimates about leg shape, reflecting the ever-present “overcomplete” feeling of being in one’s own body, but
2. underestimate their disowned/affected leg(s) (unilateral-desire) or both (bilateral-desire or paralysis-desire) legs more than that same leg of controls during the more implicit, tactile or proprioceptively guided tasks, reflecting the possibly incomplete or underdeveloped higher-order representation of the limb.

That is, if the sensory input has problems cross-referencing with a model of the legs in the brain, judgments about bodily dimensions and its position in space might be reflective of only a portion of the (total possible) input about one’s body configuration if visual input is not there to correct for it. Understanding the perceived internal configuration of the legs in BIID might provide insight into the incongruent experience they have, and eventually move towards modulating these representations to better close this gap between the perceived body and the actual body [e.g., (44–46)].

## Methods and Procedures

### Participants

#### BIID Participants

Ten participants with lower-limb BIID took part. One participant (female desiring paralysis of her legs) was removed due to motivational issues during the experiments. Therefore, nine participants were included in the current experiments. Participants were recruited via collaboration with another BIID researcher and through online BIID support group forums (https://groups.yahoo.com/neo/groups/fighting-it/info and https://forum.biid.ch/). Participants (8 biologically male) ranged in age from 19 to 68 years of age (mean = 43.1, SD = 13.5). Highest level of education completed was as follows: primary school (*n* = 1), secondary school (*n* = 1), higher education (*n* = 5), university level (*n* = 2). Two individuals desired left leg amputation, two desired right leg amputation, one desired bilateral amputation, and 4 desired bilateral paralysis of the legs. Participants had normal or corrected-to-normal vision. Tactile sensitivity was reported to be normal.

Participants took part in a telephone interview with a psychiatrist prior to their participation to confirm the desire to change the body arose from having BIID and was not a product of another psychiatric condition. We used the criteria from First and Fisher (1) to determine if the individual had BIID. In addition, questions were asked about the history of the BIID, any psychiatric illnesses, and whether they had normal tactile sensitivity and vision. See **Appendix 1**. All contacted participants were eligible for participation. For a more thorough assessment of the individual’s psychiatric profile, a trained neuropsychologist administered the Structured Clinical Interview for the DSM-5 Axis I and Axis II disorders (SCID-5) on the day of testing in Utrecht. Psychiatric profiles were overall normal for most participants. Three individuals had diagnoses prior to participation, including one with PDD-NOS, one with borderline personality disorder and post-traumatic stress disorder, and another with gender identity dysphoria (male desired to be female) which were confirmed during the clinical interview. Characteristics for each participant are included in **Table 1**.

**Table 1:** Characteristics of BIID sample. General characteristics of BIID sample. For the participant column: RA, right amputation desire; LA, left amputation desire; BA, bilateral amputation desire; P, paralysis desire. The number preceding the code is randomly assigned participant number. For the sex and gender columns: M = male, F = female. For the current comorbid conditions column: PDD-NOS, pervasive developmental disorder-not otherwise specified; BPD, borderline personality disorder; PTSS, post-traumatic stress symptoms.

Participant	Sex	Gender	Age (years)	Highest level of education obtained	Desire (lower limbs only)	Desire since age	Current comorbid conditions
**1**—**RA**	M	M	40	Higher education	Right knee disarticulation	10	PDD-NOS
**2**—**RA**	M	M	42	Secondary school	Right above knee amputation	6	none
**3**—**LA**	M	M	51	Higher education	Left above knee amputation	6	none
**4**—**LA**	M	M	42	University degree	Left above knee amputation	7	none
**5**—**BA**	M	M	38	Higher education	Bilateral above knee amputation	6	none
**6**—**P**	F	F	19	Primary school	Paralysis	6	BPD, PTSS, Dysthymia
**7**—**P**	M	F	35	Higher education	Paralysis	20	Gender Dysphoria
**8**—**P**	M	M	68	Higher education	Paralysis	6	none
**9**—**P**	M	M	51	University degree	Paralysis	10	none

#### Controls

Approximately two sex- and age-matched (± ~5 years) control participants were tested per BIID participant, so the total control group consisted of 21 participants (17 males) between the ages of 21 and 71 (mean = 44.9, SD = 15.2). Highest level of education completed was as follows: secondary school (*n* = 4), higher education (*n* = 9), university level (*n* = 8). Participants reported normal tactile sensitivity and normal or corrected-to-normal vision. All participants reported no past/current psychiatric illnesses, and this was corroborated by our screenings with the Modified MINI-screen (47) and a SCID-V questionnaire for personality disorders (48). Participants were recruited via online study participant websites, Utrecht University’s intranet, and word of mouth.

All participants gave written informed consent in accordance with the Declaration of Helsinki. The protocol was approved by the internal ethics committee of the Faculty of Social and Behavioral Sciences at Utrecht University (protocol number: FETC 17-004) before participation. Participants were naïve to the purposes of the study.

### Questionnaires

#### Demographics and Medical History

Control participants completed a general questionnaire which included questions about the demographics (e.g. age, sex, ethnicity) and medical history (e.g. presence of psychiatric or chronic medical disorder).

BIID participants completed a more extensive version of the questionnaire with additional questions about their BIID, which was a modified version of the BIID Phenomenology Questionnaire (4). These questions were administered to provide a richer understanding of the history, experience, and description of the individual’s condition.

#### 12-Item Zurich Xenomelia Scale

This scale was given to BIID participants only. This has been described by us in the supplementary material elsewhere (49). The 12-item Zurich Xenomelia Scale (ZXS) (5), consists of 3 subscales regarding 1) the strength of the participant’s amputation (or paralysis) desire, 2) the participant’s erotic attraction to amputees/being an amputee, and 3) the extent to which the participant engages in pretending behaviors (i.e. simulated the bodily state of being amputated or paralyzed). Participants rated their agreement with each statement from 1 (strongly agree) to 6 (strongly disagree). Items 1 (reverse-scored), 2, 5 (reverse-scored), 10 are part of the “pure amputation (paralysis) subscale,” items 3, 6(reverse-scored), 9 (reverse-scored), 12 are part of “erotic attraction” and items 4, 7, 8 (reverse-scored), 11 (reverse-scored) are part of the “pretending behavior” subscale. We modified the ZXS to accommodate all participants, i.e., “amputation” was replaced with “paralysis” for participants who desire paralysis.

#### Sheehan Disability Scale (SDS)

Note: this was given to BIID participants only.

The SDS is a scale which assesses functional impairment in work/school, social, and family life due to having a disability or impairment, in this case BIID (50). Participant rated their agreement [from 0 (not at all) to 10 (extremely)] with three statements about the extent to which their BIID symptoms have disrupted work/school, social life, and family life in the past week. The number of days in the past week that were lost and that were underproductive were also recorded.

### Body Representation Tasks

Methods were similar to Stone et al. (33). Order of tasks was counterbalanced between participants with the BIID group. Participants in the control group received the same task order as the BIID participant they were matched to.

#### Template Matching Task (Visual Body Representation)

**Figure 1:**
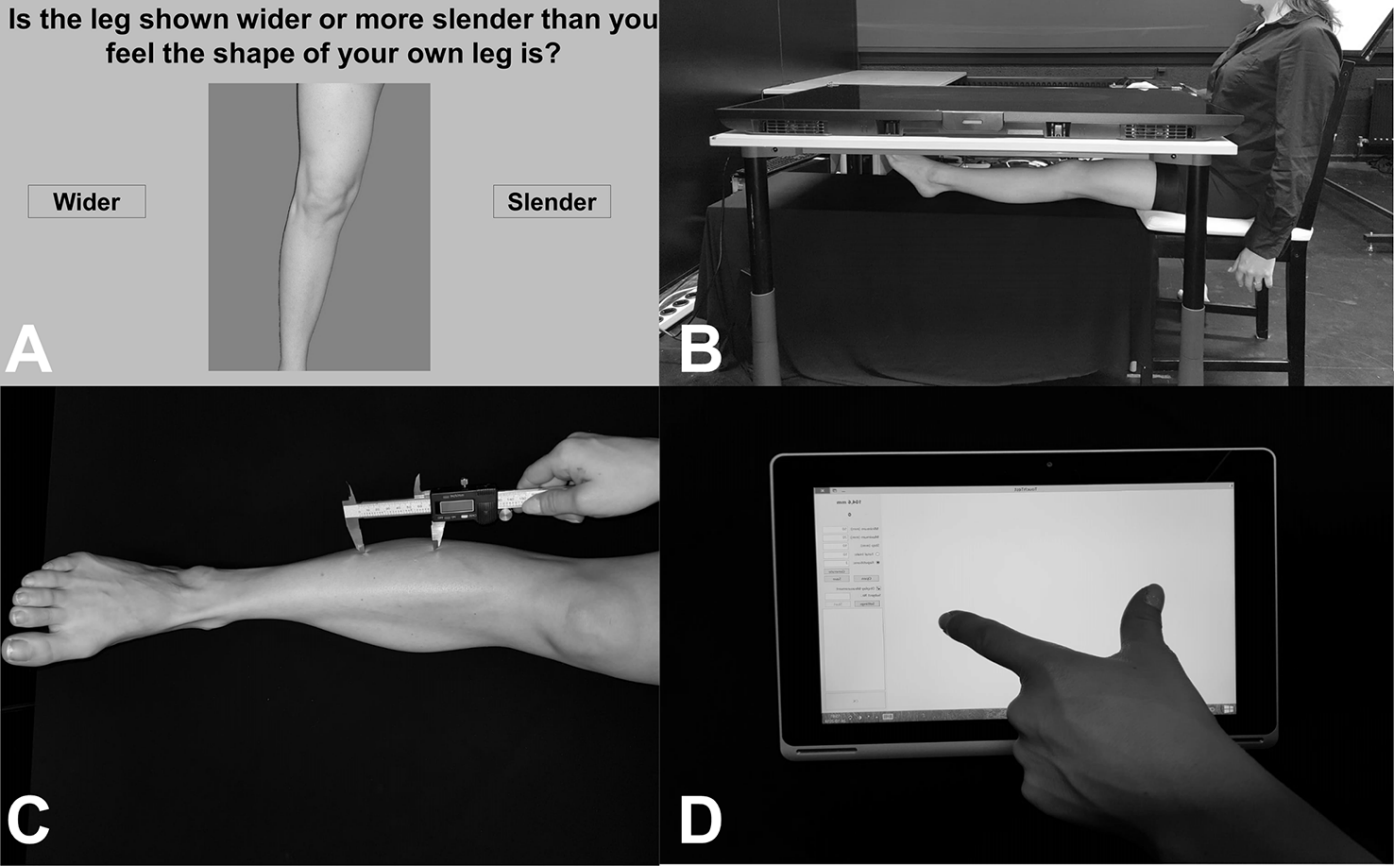
**(A)** Image showing an example trial from the Template Matching Task. Participants were presented with distorted images of their own legs and asked to judge whether the leg was wider/slender (or longer/shorter, not pictured here) than their own leg by clicking one of the buttons on either side of the image. **(B)** Image showing setup for localization task, in an example trial of the Real condition. Participants placed their legs under a television screen and were asked to click-to-indicate (using a mouse in their right hand, not pictured here) the position of different leg landmarks. **(C)** Image showing an example trial from the tactile distance estimation task. Two unseen points were applied to the leg in the vertical (pictured here) or horizontal direction. **(D)** Image showing example response for the tactile distance estimation task. While blindfolded, participants judged the distanced between the two applied points (e.g. as shown in **C**.) using their thumb and index finger on a touchscreen tablet.

Body representations about the visual properties of the legs were assessed using a Template Matching Task (29, 33, 51). Participants made judgments about the length/width of their body parts in a custom-made MATLAB program. Photographs were taken of the participants’ bare legs (mid-thigh to ankle), which were then entered into a custom-made MATLAB program. The program generated fifteen images ranging in size from 65% to 135% (in increments of 5%) of the image’s length *or* width. These images were displayed, one at a time, on a vertically positioned computer monitor (27 L × 34 W; resolution: 1280 × 1024). For the images that were stretched horizontally, participants indicated, by clicking one of two onscreen buttons, whether the image of the leg shown onscreen was wider or more slender than he/she felt the shape of his/her own leg was. For images that were stretched vertically, participants were asked to choose whether image of the leg shown onscreen was shorter or longer than he/she felt the shape of his/her own leg. The program used a staircase procedure to determine the participants’ perceived leg width/length [described in greater detail in (33)]. The average value of the last five reversals was taken as the perceived size (range of values 0.65–1.35). For example, an average value of 1.22 on the length estimates would indicate that the participant perceived his leg to be 122% of its actual image size, or 22% longer. Starting condition (length right, length leg, width right, width left) was counterbalanced between participants. See **Figure 1A** for visualization of an example trial.

#### Tactile Distance Estimation Task (Tactile Body Representation)

Body representation underlying tactile distances was assessed using the tactile distance estimation task (33, 39). A digital caliper was used to apply two points with a pre-specified distance (for legs: 50, 60, or 70 mm; for arms: 40, 50, 60 mm) to the participant’s body part in the horizontal (width-wide, medio-lateral) or vertical (length-wise, proximo-distal) direction. The difference in distances were due to the 2-point discrimination threshold for the shin [e.g. 45 mm, (52)] and the small size of some individuals’ arms (i.e. applying 70 mm extends the width of some people’s arms). The reason for this directional manipulation was to assess biases in tactile direction estimates, which have been shown before (33, 53, 54). While blindfolded, participants estimated the distance between those two points by mimicking the distance with their right thumb and index finger and placing it on an ACER Aspire 10-inch tablet. A custom-made program (TouchTest, programmed in MATLAB) recorded the responses. As in Stone et al. (33), each distance was applied three times per location (shin, forearm, thigh), side (right, leg), and direction (horizontal, vertical). In total, 18 stimuli were applied per body part. Starting condition based on location and direction were counterbalanced between participants. See **Figures 1C, D** for example of setup and response.

##### Controls

Stimuli were applied to all six parts (left shin, right shin, left thigh, right thigh, left forearm, right forearm). However, it is important to note that only participant 5-BA received the task on his thighs, and therefore is the only participant compared to this condition.

##### BIID Participants

###### Amputation Variant

To assess tactile distances on affected versus unaffected parts of the body, stimuli were applied to two parts (right shin, left shin). In all unilateral-desire cases, one shin was coded as an affected part. Participant 5-BA, who desired bilateral amputation, received tactile stimuli above (thigh) and below (shin) the demarcation line, such that the shins constituted the affected parts.

###### Paralysis Variant

As participants desired paralysis of their legs from the waist down, we used the forearm as an “unaffected” body part. Distances were thus applied to the four parts (left shin, right shin, left forearm, right forearm). In all cases, both shins were coded as affected parts.

#### Localization Task (Proprioceptive and Other Body Representations)

Leg representations underlying proprioception, proprioceptive imagery, and visual memory were assessed using the Leg Localization Task (33), employed several times for the hands: [e.g. (13–15, 18, 19)]. The widths of the participants’ knees, ankles, and mid-thighs (mid-point between the groin and knee) and the lengths of the participants’ upper legs (mid-thigh to knee) and lower legs (knee to ankle) were measured with a Vernier caliper (brand: MIB, 300 mm), measuring tape, and a curved caliper. Marks were made at these same locations (inner/outer mid-thigh, inner/outer knee, inner/outer ankle) using a washable marker to familiarize the participant with these landmarks.

Participants sat with their torsos centrally aligned at the short edge of a 55-inch SONY KDL-55W805C television (screen dimensions: 68.5 cm W (short edge) × 121.5 cm L (long edge); resolution: 1920 × 1080) which lay horizontally on a tabletop (120 L × 80 W × 70 H cm). Text indicating 1 of 6 landmarks (inner mid-thigh, outer mid-thigh, inner knee, outer knee, inner ankle, outer ankle) was presented adjacent to the participant on the other end of the screen for each trial. Using a mouse in the right hand (positioned at the same height as the table), participants moved the cursor to the perceived location of the presented landmarks and clicked to indicate its position under the following conditions:

- Real: participants outstretched their legs on a tabletop (100 L × 60 W × 44.5 H cm) located 30 cm below the television, rendering it out of sight. To prevent movement during each block, the heel was positioned to rest on a foam pad. Participants were asked to indicate the felt position of that landmark by left-clicking directly above where they *felt* (i.e. relying on proprioceptive feedback) that part of their leg to be. This was completed twice, once for each leg. See **Figure 1B** for example of this condition.
- Imagine: participants were instructed to “imagine as though your leg is extended under the table” while sitting normally at the setup (legs bent at 90 degrees). Participants therefore relied on proprioceptive imagery (33, 55), rather than proprioceptive feedback, to complete the task. This was completed twice, once for each leg.
- Mannequin: participants were instructed to indicate the same landmarks on a mannequin leg. They studied a right mannequin leg, with the same marked landmarks as their own legs, for 30 s and were urged to memorize its shape and landmarks. The mannequin leg was then placed under the television screen on the lower tabletop. Participants sat normally, as in the Imagine condition.

A custom-made MATLAB program was used to display the text and record the data. Participants completed 60 randomized trials (10 trials per landmark) per condition. Starting condition (real right, real left, imagine right, imagine left, mannequin) was counterbalanced between participants. To familiarize participants with the task, a short 12-trial practice block was completed before the experiment started (data not included in analysis). See **Figure 1B** for example of setup.

### Data Analysis

#### Template Matching Task

The average value for each image was taken as the last 5 reversals across both staircases for each image. Average values could range from 0.65 to 1.35 as images shown were between 65% to 135% of the length or width of the body part’s image. For controls, a one-sample t-test comparing the values to 1 (veridical performance) was used to examine whether participants over- or under-estimated the shapes of their legs/feet.

The average estimates for each part were individually compared between BIID participants and controls.

#### Tactile Estimation Task

For controls, the average estimates per distance were calculated by taking the average of the three trials per distance. Separate repeated measures ANOVA with distance and body as the within-subject factors were conducted to examine if participants estimated larger distances for 70 mm vs 50 mm vs 60 mm, for example for each part. There was a main effect of distance, showing that participants estimated distances as larger as the applied distance was indeed larger. There was no interaction between distance and body part. Therefore, we collapsed across distance estimates, and converted it to a percent mis-estimation for each body part and direction (i.e. %mis-estimation = (perceived distance-actual average distance)/actual average distance). To compare performance, distances were collapsed for BIID participants as well. See supplementary material (**Figures S1–S4**) for plots displaying judgments per distance, per participant for controls and BIID participants.

Comparison to Amputation Variant: Percent mis-estimations for the affected and unaffected shins were compared to the shins of controls. For participant 5-BA (bilateral-amputation desire), percent mis-estimations for the affected shins (both) and the unaffected (thigh) were compared to the shins and thighs of controls.

Comparison to Paralysis Variant: Percent mis-estimations for the shins (both affected) were compared to the shins of controls. Percent mis-estimations for the forearms (both unaffected) were compared to the forearms of controls.

#### Localization Task

On-screen coordinates were compared to the actual dimensions of the leg. Each pixel on screen represented 0.63 mm. For each landmark, the average x/y coordinates of the 10 clicked points per landmark was calculated. Points > 2 SDs from the average clicked location were removed.

The perceived distance between the two points (e.g. inner knee to outer knee) were calculated using the equation:

$$d=\sqrt{{\left({\text{x}}_{2}–x_{1}\right)}^{2}+{\left(y_{2}–y_{1}\right)}^{2}}$$

To convert from pixels to mm, “d” was multiplied by 0.63 (due to screen size and resolution). The perceived and actual shapes of the lower legs were calculated in order to compare the BIID legs to control legs (33). This is a measure of the overall aspect ratio of an object/body part (34, 56), which can be calculated as 100 * width/length. As at least one lower leg (knee to ankle) was affected in all BIID participants, we calculated lower leg (perceived and actual) SIs using the following equation:

SI=100∗average of ankle and knee widths (in mm)/length from knee to ankle (inmm)

A value of 100 would suggest that width is equal to length (i.e. a square shape). A value > 100 would suggest that width > length (i.e., a shape that is shorter than it is long) and <100 would suggest that width < length (i.e. a shape that is longer than it is wide). Importantly, actual SIs for the lower leg are <100 as the width of the ankle and knees are always less than the length of the shin bone in normal legs. However, perceived SIs can entertain all possible outcomes (e.g. I could perceive my leg to be shorter than it is long). To compare across conditions in the localization task, we used these actual and perceived SIs to then calculate a *normalized* shape index (NSI = perceived SI/actual SI) to use as an outcome measure for each participant and condition. Values of 1 indicated that participants accurately perceived the shape of the leg part. Values > 1 indicated that the perceived shape of the leg was shorter and/or wider than the actual shape (i.e. the perceived proportion of width to length is higher than the actual proportion of width to length, or in other words, a foreshortened leg length with respect to its width). Conversely, values < 1 indicated that the perceived shape of the leg was longer and/or thinner than the actual shape (i.e. the perceived proportion of width to length is lower than the actual proportion of width to length, or in other words, a lengthened leg with respect to its width). NSIs were compared between BIID participants and controls.

*BIID versus Controls:* Due to the heterogeneity of the BIID sample (e.g. sex, affected body part, variant of BIID, age), we took a multiple single-case approach, comparing each BIID participant separately to the control group. Outcomes for each task were compared between BIID subjects and controls using Crawford-Garthwaite Bayesian single-case t-tests (57) in R using the psycho package (58), one-sided (as we hypothesized an *under*estimation of leg size for the localization (NSIs > 1) and tactile distance estimations (% mis-estimation < 0), but an *over*estimation of leg size for the template matching task compared to controls). Bonferroni-corrections for multiple comparisons were used when necessary. Microsoft Excel 2016 was used to process and visualize the data. IBM SPSS Statistics 23.0 for Windows (IBM Corp., Armonk, N.Y., USA) and JASP [version 0.9.2.0, (59)] were also used for analysis/further visualization of the data. For clarity, only the lowest *p* value is stated for the multiple single case comparisons. Tables including all *p* values, credible intervals, and effect sizes for each BIID participant on each outcome measure per task in comparison to controls is included in the **Supplementary Material**.
Outlier Analysis: In total, control participants with scores > 2.5 SDs from the mean score were removed from the analysis. One control participant was removed from the tactile estimation task and two control participants were removed from the localization task. One participant did not complete the arm trials for the tactile estimation task but completed the task for the legs. Analyses were done within tasks.

## Results

### Questionnaires

#### 12-Item Zurich Xenomelia Scale

Average scores ± standard deviations for each subscale were as follows: 5.4 ± 0.6 (pure amputation/paralysis desire), 3.2 ± 1.3 (erotic attraction), and 4.7 ± 0.6 (pretending behaviors). Total average score was 4.4 ± 0.8 out of a possible 6. These scores are in line with previous studies using this scale to describe their BIID sample [e.g. (5, 11, 21)]. See **Table 2**.

**Table 2:** 12-item Zurich Xenomelia (ZXS) scores per BIID participant.

**Participant**	**ZXS:** **Total Score**	**Pure amputation/paralysis desire subscale**	**Erotic attraction** **subscale**	**Pretending behaviors** **subscale**
**1 - RA**	4.42	4.75	3.5	5
**2 – RA**	4.83	5.5	4.25	4.75
**3 – LA**	3.83	5.5	1.5	4.5
**4 – LA**	5.50	6	5	5.5
**5 – BA**	4.75	6	4.75	3.5
**6 – P**	4.25	6	2	4.75
**7 – P**	3.92	4	2	5.75
**8 – P**	4.08	5.5	2.25	4.5
**9 – P**	4.75	5.75	4	4.5

The total score is the average of all scores. Scores could range from 1 to 6, with higher scores indicating stronger BIID symptomatology.

#### Sheehan Disability Scale (SDS)

Average scores ± standard deviations for each subscale (out of a total possible score of 10) were as follows: 6.5 ± 2.6 (work/school life), 6.6 ± 1.9 (social life), 5.1 ± 2.9 (family life). Values between 6 to 7 reflect *moderate to marked* disruption. Values between 5 to 6 reflect *marked* disruption (50). The average number of days lost due to BIID was 0.4 ± 0.7 days. The average numbers of underproductive days due to BIID was 1.6 ± 2.0.

### Body Representation Tasks

#### Template Matching Task

##### Controls

Controls were accurate in making judgments about right leg width (*t*(20) = 1.5, *p* = 0.2), right leg length (*t*(20) = 1.8, *p* = 0.08), left leg length (*t*(20) = 1.6, *p* = 0.1), and left leg width (*t*(20) = 1.8, *p* = 0.07) as revealed by a series of one-sample t-tests comparing the scores to 1 (i.e. veridical performance).

**Figure 2:**
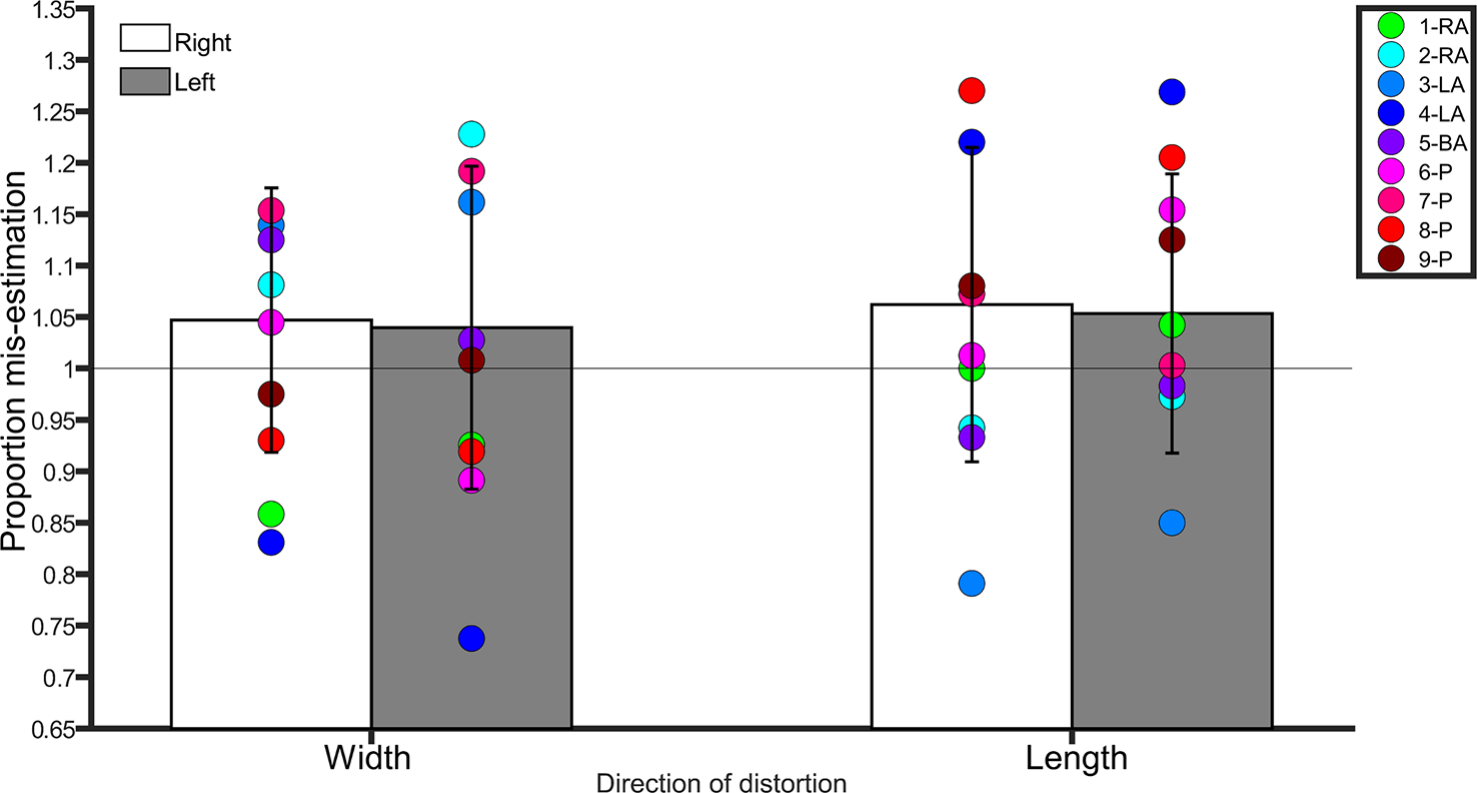
Bar graph showing proportion mis-estimation (1 is veridical) for judgments of images distorted width-wise or length-wise in the Template Matching Task. The white bars represent estimates of the right leg in controls. The grey bars represent estimates of the left leg in controls. Error bars represent standard deviation. The horizontal line positioned at y = 1 denotes veridical performance on this task. Each colored point represents a BIID participant. RA, right amputation; LA, left amputation; BA, bilateral amputation; P, paralysis in the legend. The preceding numbers denotes participant number.

A repeated-measures ANOVA with side (left, right) and direction of distortion (width, length) as within-subjects factors conducted on the average scores revealed no main effect of side (*F*(1,20) = 0.3, *p* = 0.5, η2 = 0.01), direction (*F*(1,20) = 0.08, *p* = 0.7, η2 = 0.004), and no interaction between side and direction (*F*(1,20) = 0.005, *p* = 0.9, η2 < 0.0001). Controls were overall accurate in making judgments about the length and width of images of their legs, regardless of side. See **Figure 2**.

##### Comparison to BIID Participants

###### Right

*Width*. There was no difference between BIID participants and controls (*p* ≥ 0.1 for remaining comparisons). *Length*. No differences between BIID participants and controls emerged (*p* ≥ 0.07 for remaining comparisons, and *p* = 0.04 for participant 3-LA due to underestimation but irrelevant due to one-tailed testing).

###### Left

*Width*. No differences between BIID participants and controls emerged (*p* ≥ 0.1 for remaining comparisons, and *p* = 0.01 for participant 7-LA due to underestimation but irrelevant due to one-tailed testing). *Length.* No differences between BIID participants and controls emerged (*p* ≥ 0.1 for remaining comparisons). See **Figure 2** for individual results.

Therefore, our hypothesis that participants with BIID would overestimate the size of their affected legs more than the same leg of controls, mimicking the conscious experience of being “overcomplete,” was not confirmed. See **Tables S1.1–S1.4** in supplementary material for all p-values, confidence intervals, and effect sizes for BIID participants compared to controls.

#### Tactile Estimation Task

##### Controls (Raw Estimated Values)

###### Shin

A repeated-measures ANOVA with distance (50, 60, 70 mm), side (left, right), and direction (horizontal, vertical) revealed a main effect of direction, indicating that participants judged distances applied in vertical direction as smaller than those same distances applied in the horizontal direction (*F*(1,19) = 28.0, *p* < 0.0001, η2 = 0.5). There was a main effect of distance, indicating that participants judged larger distances as larger, e.g. 60 mm > 50 mm (*F*(2,38) = 43.3, *p* < 0.0001, η2 = 0.6). There was no main effect of side, indicating that estimates were similar for left and right legs (*F*(1,19) = 0.1, *p* = 0.7, η2 = 0.008). No interactions were significant (*p* ≥ 0.3 for all comparisons).

###### Arm

A repeated-measures ANOVA with distance (40, 50, 60 mm), side (left, right), and direction (horizontal, vertical) revealed a main effect of direction, indicating that participants judged distances applied in vertical direction as smaller than those same distances applied in the horizontal direction (*F*(1,18) = 51.5, *p* < 0.0001, η2 = 0.7). There was a main effect of distance, indicating that participants judged larger distances as larger, e.g. 60 mm > 50 mm (*F*(2,36) = 48.8, *p* < 0.0001, η2 = 0.7). There was no main effect of side (*F*(1,18) = 1.6, *p* = 0.2, η2 = 0.08). The interaction between distance and direction was significant (*F*(1.4, 26.8) = 5.7, *p* = 0.01, η2 = 0.2). Paired samples t-tests (where the critical Bonferroni-correct *p* = 0.016) revealed that 60 mm were judged as larger than 50 mm (and 50 mm larger than 40 mm) for the horizontal directions (*p* < 0.0001 for all), but 60-mm distances applied in the vertical direction failed to be judged as larger than 50-mm distances in the vertical direction, thereby showing a slightly less pronounced upward step in distance estimates (*t*(18) = −2.3, *p* = 0.057). No other interactions were significant (*p* ≥ 0.2 for all comparisons).

###### Thigh

A repeated-measures ANOVA with distance (50, 60, 70 mm), side (left, right), and direction (horizontal, vertical) revealed a main effect of distance, indicating that participants judged larger distances as larger (*F*(1.5,27.0) = 39.4, *p* < 0.0001, η2 = 0.6). There was no main effect of side (*F*(1,18) = 1.5, *p* = 0.2, η2 = 0.07) or direction (*F*(1,18) = 0.6, *p* = 0.4, η2 = 0.03). No interactions were significant (*p* ≥ 0.5 for all comparisons).

For clarity’s sake, we collapsed across the variable “distance” for both “sides” and “directions” for the arm, thigh, and shin separately. Percent mis-estimations for each side and direction were calculated as: (average estimated distance – average applied distance)/(average applied distance) * 100. These values were used to compare to BIID participants for each condition.

##### Controls (Percent Mis-Estimation Values)

###### Shin

**Figure 3:**
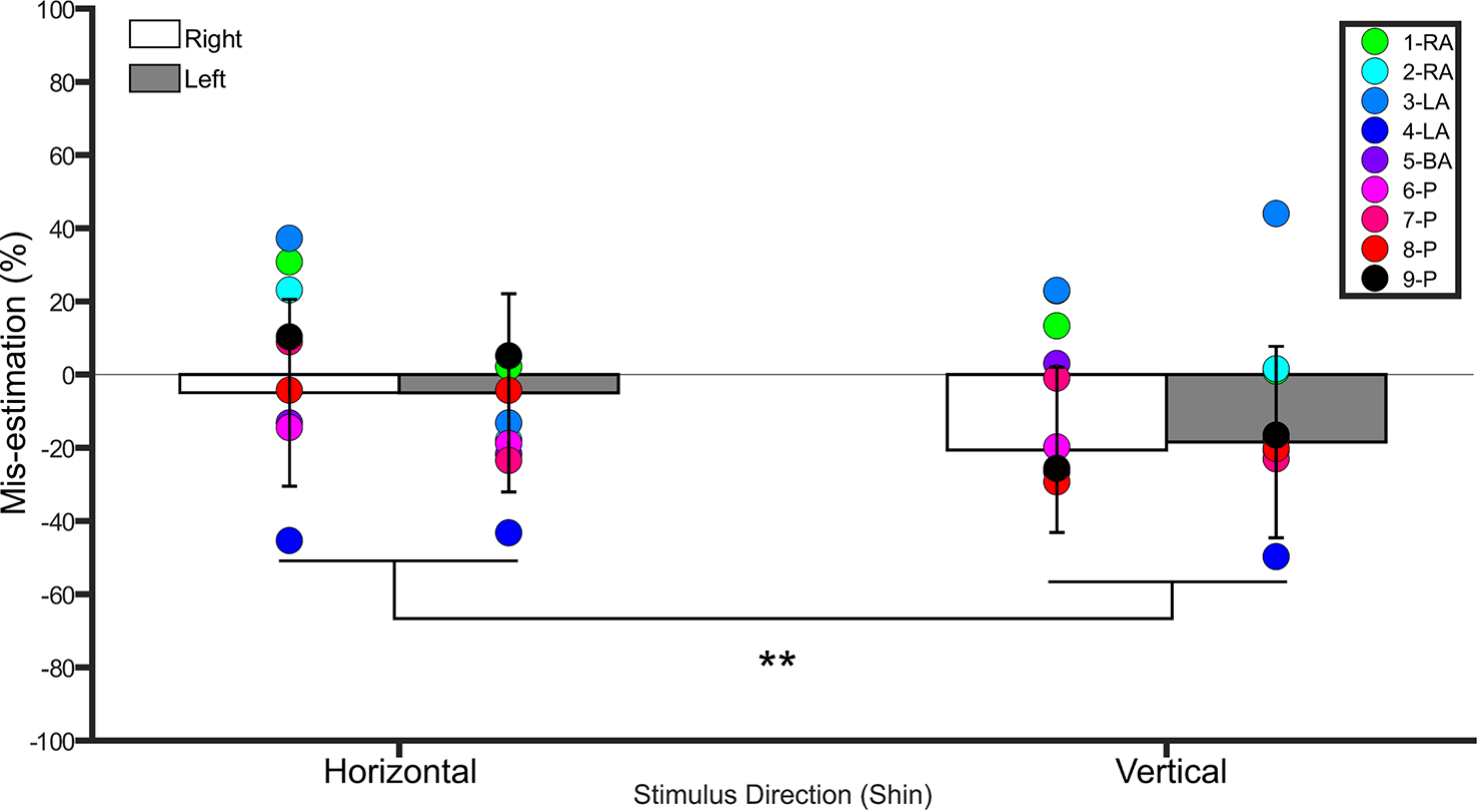
Bar graph showing percent mis-estimation of distances applied horizontally (width-wise) and vertically (length-wise) to the shins. The white bars represent percent mis-estimation for the right shin in controls. The grey bars represent percent mis-estimation for the left shin in controls. Error bars represent standard deviation. Negative values suggest under-estimation of distances applied and positive values suggest over-estimation of distances applied. Individual colored point represents percent mis-estimations for each BIID participant. RA, right amputation; LA, left amputation; BA, bilateral amputation; P, paralysis in the legend. The preceding numbers denotes participant number. Note that some estimations were so similar in BIID participants that their points overlapped. **p < 0.001 and denotes that control participants overestimated more for distances applied horizontally versus vertically.

A repeated measures ANOVA on the percent mis-estimation values with side and direction as within-subject factors revealed a main effect of direction (*F*(1,19) = 28.0, *p* <0.001, η2 = 0.5), indicating that controls underestimated distances applied in the vertical direction (−19.5% ± 23.5 SD) more than those applied in the horizontal direction (−4.9% ± 24.7 SD). There was no main effect of side (*F*(1,19) = 0.1, *p* = 0.7, η2 = 0.008). The interaction between side and direction was not significant (*F*(1,19) = 0.2, *p* = 0.6, η2 = 0.01). One-sample t-tests comparing the values to 0 (Bonferroni-corrected *p* = 0.01) for each condition revealed that distances applied to the right (*t*(19) = −4.0, *p* < 0.001) and left (*t*(19) = - 3.1, *p* = 0.005) shins in the vertical direction were significantly different from 0, suggesting that these distances were underestimated. Distances applied in the horizontal direction were not different from 0 (*p* ≥ 0.3 for both comparisons). See **Figure 3**.

###### Arm

**Figure 4:**
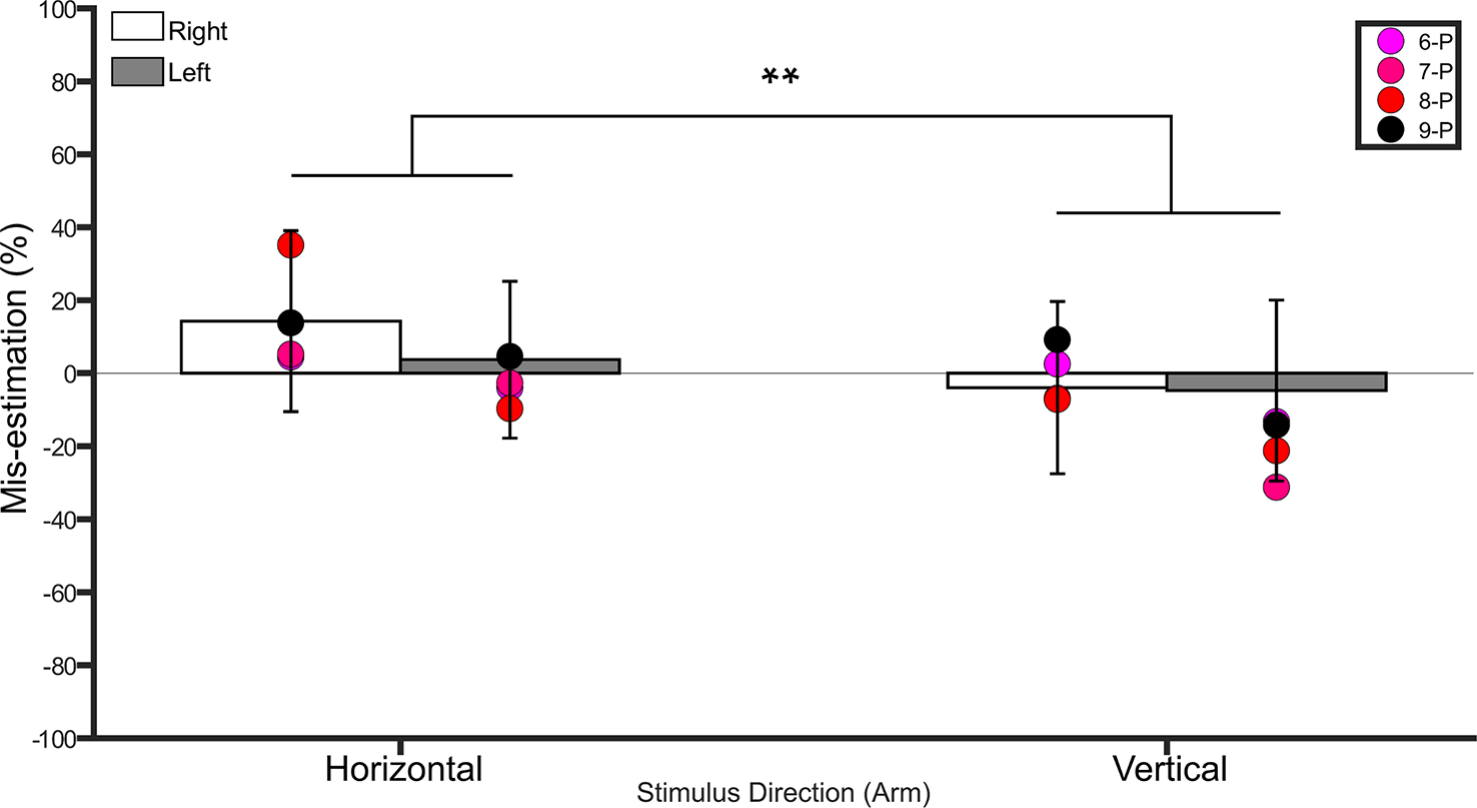
Bar graph showing percent mis-estimation of distances applied horizontally (width-wise) and vertically (length-wise) to the forearms. The white bars represent percent mis-estimation for the right forearm in controls. The grey bars represent percent mis-estimation for the left forearm in controls. Error bars represent standard deviation. Negative values suggest under-estimation of distances applied and positive values suggest over-estimation of distances applied. Individual colored point represents percent mis-estimations for each BIID participant. P = paralysis in the legend. The preceding numbers denotes participant number. Note that some estimations were so similar in BIID participants that their points overlapped. **p < 0.001 and denotes that control participants overestimated more for distances applied horizontally versus vertically.

A repeated measures ANOVA on the percent mis-estimation values with side and direction as within-subject factors revealed a main effect of direction (*F*(1,18) = 51.4, *p* <0.001, η2 = 0.7), indicating that controls underestimated distances applied in the vertical direction (−4.3% ± 21.3 SD) more than those applied in the horizontal direction (8.9% ± 21.1 SD). There was no main effect of side (*F*(1,18) = 1.6, *p* = 0.2, η2 = 0.08). The interaction between side and direction was not significant (*F*(1,18) = 2.9, *p* = 0.1, η2 = 0.1). One-sample t-tests comparing the values to 0 (Bonferroni-corrected *p* = 0.01) for each condition revealed no difference from 0 (i.e. veridical performance, *p* ≥ 0.4 for left horizontal, left vertical, and right vertical, *p* = 0.029 for right horizontal). See **Figure 4**.

###### Thigh

A repeated measures ANOVA on the percent mis-estimation values with side and direction as within-subject factors revealed no main effect of direction (*F*(1,18) = 1.5, *p* = 0.2, η2 = 0.07), no main effect of side (*F*(1,18) = 0.6, *p* = 0.4, η2 = 0.03), nor an interaction between side and direction (*F*(1,18) = 0.2, *p* = 0.6, η2 = 0.01). One-sample t-tests comparing the values to 0 (Bonferroni-corrected *p* = 0.01) was significant for all comparisons (right horizontal: *p* = 0.002, left horizontal: *p* = 0.01, right vertical: *p* < 0.001, left vertical: *p* = 0.006).

##### Comparison to BIID

###### Shin

The shins were tested on all participants. Both shins were “affected” in paralysis-variant participants and the bilateral-amputation desire participant. One shin was “affected” in unilateral amputation desire participants (i.e. 2 left, 2 right). See **Figure 3** for individual results.

###### Right

*Horizontal*. BIID participants did not significantly underestimate distances more than controls (*p* ≥ 0.06 for all comparisons). *Vertical*. BIID participants did not significantly underestimate distances more than controls (*p* ≥ 0.08 for all remaining comparisons, except *p* = 0.04 for participants 2-RA and 3-LA but in the opposite direction, i.e. overestimation, irrelevant due to one-tailed testing).

###### Left

*Horizontal*. BIID participants did not significantly underestimate distances more than controls (*p* > 0.09 for all comparisons). *Vertical.* BIID participants did not significantly underestimate distances more than controls (*p* ≥ 0.1, except *p* = 0.02 for participant 3-LA but in opposite direction, i.e. overestimation, irrelevant due to one-tailed testing).

Therefore, our hypothesis that participants with BIID would underestimate distances applied to their affected parts more than controls was not confirmed. See **Tables S2.1–S2.4** in supplementary material for all p-values, confidence intervals, and effect sizes for BIID participants compared to controls.

###### Arm

The arms were only tested on participants who desired paralysis, as the arms served as the unaffected part. See **Figure 4** for individual results.

###### Right

*Horizontal*. No differences between BIID and control participants emerged (*p* ≥ 0.2 for remaining comparisons). *Vertical*. No differences between BIID and control participants emerged (*p* ≥ 0.3 for all comparisons)

###### Left

*Horizontal*. No differences between BIID and control participants emerged (*p* ≥ 0.3 for all comparisons). *Vertical*. No differences between BIID and control participants emerged (*p* ≥ 0.1 for all comparisons).

See **Tables S2.5–S2.8** in supplementary material for all p-values, confidence intervals, and effect sizes for BIID participants compared to controls.

##### Thigh (Unaffected Site for Participant 5-BA)

###### Right

*Horizontal*. There was no difference between participant 5 (7.0%) and controls (−20% ± 25 SD; *p* = 0.1). *Vertical.* There was no difference between participant 5 (−33.8%) and controls (−21.3% ± 23.2 SD; *p* = 0.3).

###### Left

*Horizontal*. There was no difference between participant 5 (−21.9%) and controls (−16.4% ± 24.7 SD; *p* = 0.4). *Vertical.* There was no difference between participant 5 (0.5%) and controls (−20.2 ± 28.0 SD; *p* = 0.2).

See **Tables S2.9–S2.12** in supplementary material for all p-values, confidence intervals, and effect sizes for BIID participants compared to controls.

#### Localization Task

NSIs were compared between BIID and control participants for the lower legs (knees to shins).

##### Controls

###### Comparison to 1 (Veridical Performance)

NSIs for all conditions were significantly different from 1 (*p* < 0.001 for all comparisons), such that they were greater than 1. Importantly, an NSI >1 indicates that the participant perceives the proportion of width to length of the lower leg as *larger* than the actual proportion, suggesting a foreshortened leg shape.

**Figure 5:**
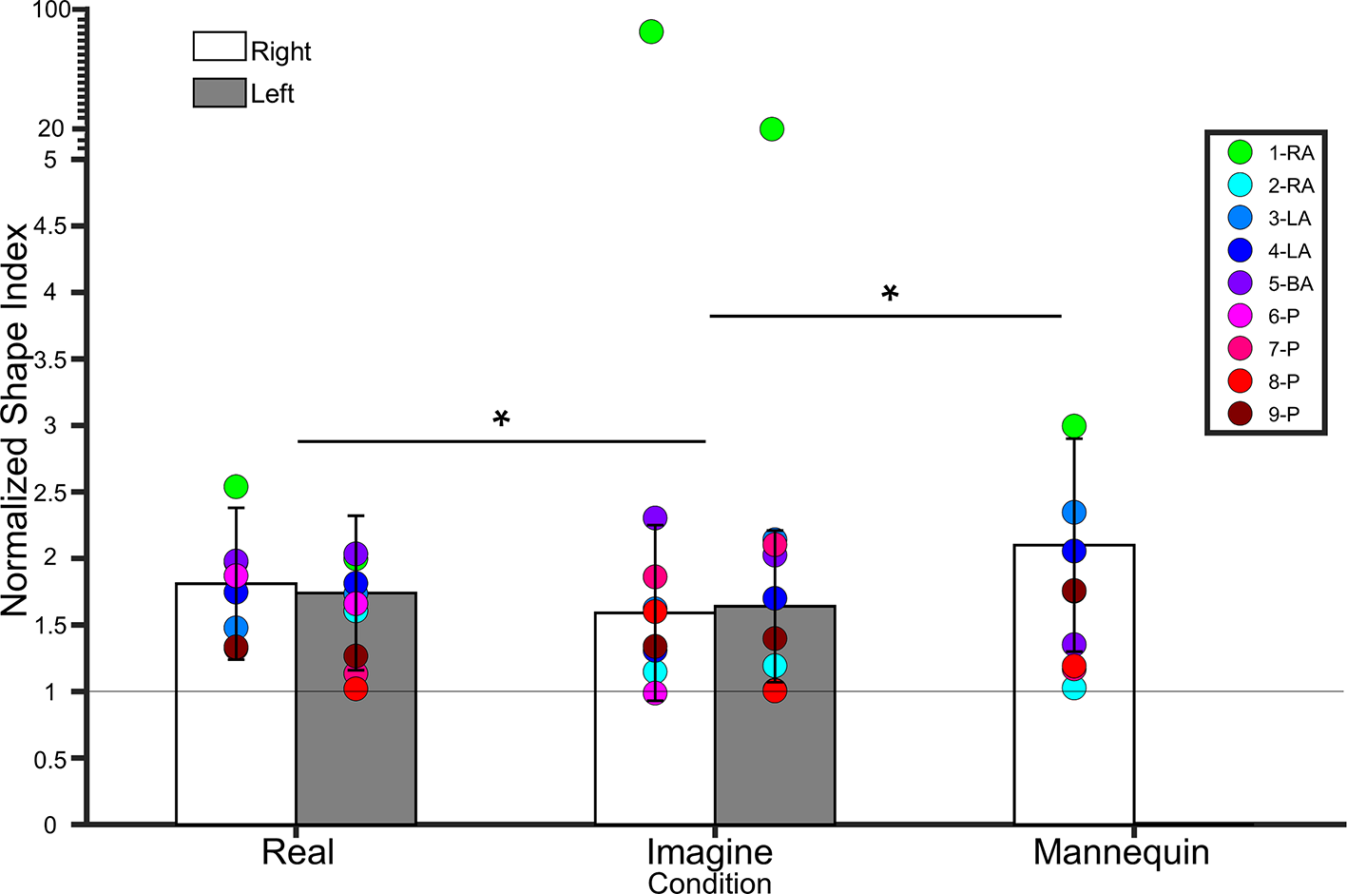
Bar graph showing lower leg normalized shape indices (NSI). The white bars represent NSIs for the right shin in controls. The grey bars represent NSIs for the left shin in controls. Error bars represent standard deviation. The grey horizontal bar at y = 1 denotes veridical shape perception of the lower leg. Individual colored point represents lower leg NSIs for each BIID participant. RA, right amputation; LA, left amputation; BA, bilateral amputation; P, paralysis in the legend. The preceding numbers denotes participant number. Note that some estimations were so similar in BIID participants that their points overlapped. *p < 0.05 and denotes that, for control participants, NSIs in the Real condition were significantly higher than NSIs in the Imagine condition, and NSIs in the Mannequin condition were significantly higher than NSIS in the Imagine condition (regardless of leg). Note: miniature y-ticks following NSI of 5 each correspond to a value of 5 in order to display extremely high NSIs from participant 1 (i.e. NSI Right leg Imagine = 86.9; NSI Left leg Imagine = 20.8).

*Own legs*. A 2 (leg) × 2 (condition) repeated measures ANOVA on lower leg NSIs revealed a main effect of condition (*F*(1,18) = 4.9, *p* = 0.03, η² = 0.2), indicating that NSIs were higher for the Real (1.77 ± 0.50 SD) condition than the Imagine (1.62 ± 0.54 SD) condition. There was no main effect of Leg (*F*(1,18) = 0.006, *p* = 0.9, η² < 0.001), nor an interaction between condition and leg (*F*(1,18) = 0.9, *p* = 0.3, η² = 0.04). See **Figure 5**.

###### Comparison with mannequin condition

To compare to the mannequin leg condition (for which we only tested a right mannequin leg), we collapsed across side for participants’ own legs and ran a repeated measures ANOVA on condition (real, imagine, mannequin). There was a main effect of condition (*F*(1.2,22.7) = 5.8, *p* = 0.01, η² = 0.2). Bonferroni-corrected pairwise comparisons revealed a significant difference between Mannequin (2.1 ± 0.8 SD) and Imagine conditions (*t*(19) = 2.88, *p* = 0.01), but not Mannequin and Real (*t*(18) = 1.9, *p* = 0.07). The difference between Real and Imagine was also significant (*t*(19) = 2.2, *p* = 0.03). See **Figure 5**.

##### Comparison to BIID

Each condition (Right Real, Left Real, Right Imagine, Left Imagine, Mannequin) were compared to BIID participants separately. See **Figure 5** for individual results.

###### Right

*Real*. No differences between BIID and control participants emerged (*p ≥* 0.1 for all comparisons). *Imagine*. Participant 1-RA scored significantly higher than controls (NSI = 86.9, *p* < 0.0001). No other differences between BIID and control participants emerged (*p ≥* 0.1 for all comparisons).

###### Left

*Real*. No differences between BIID and control participants emerged (*p ≥* 0.1 for all comparisons). *Imagine*. Participant 1-RA scored significantly higher than controls (NSI = 20.8, *p* < 0.0001). No other differences between BIID and control participants emerged (*p ≥* 0.1 for all comparisons).

###### Mannequin

No differences between BIID and control participants emerged (*p ≥* 0.1 for all comparisons).

Therefore, our hypothesis that participants with BIID would have a higher NSI (more distorted, foreshortened perceptual representation of the affected leg(s)) was not confirmed. See **Tables S3.1–S3.5** in supplementary material for all p-values, confidence intervals, and effect sizes for BIID participants compared to controls.

## Discussion

In the current study we behaviorally investigated lower limb representations in individuals with and without Body Integrity Identity Disorder (BIID), a rare condition wherein individuals desire to be disabled (1). Most commonly, this involves the desire to amputate or paralyze one or more perfectly healthy limb(s) (4). In a series of behavioral tasks, participants with and without the desire to amputate or paralyze their legs were asked to make judgments about the size and shape of their legs when relying chiefly on vision, touch, or proprioception. We replicated previous findings in our control sample, such that healthy participants were overall accurate at making judgments about visual representations of their legs, but showed systematic distortions for estimating the metrics underlying the tactile and proprioceptive representations of the legs (27, for hands see: 33, 37, 60). We hypothesized that individuals with BIID over/underestimate the metric representations of their legs more than healthy controls, as their internal representation of their legs, at some level of multimodal integration, seems to be stunted/missing (but paradoxically, at the same time people with BIID feel “overcomplete”). Using a multiple single-case analysis, we found no global differences between BIID participants and controls on any of the leg perception tasks. This suggests that despite the mismatch between the (internal) felt image of the legs and the (external) actual physical presence of the legs, individuals with BIID exhibit normal (albeit also distorted) perceptions of their legs.

Visual perception of the lower limbs was tested by asking participants to make visually-guided judgments of the length/width of distorted images of their own legs onscreen. All participants were accurate in making judgments about the size and shape of legs. These findings are in line with others that have shown accurate performance in making explicit judgments about body size when visual feedback is available [(e.g. 33)]. These results also validate the non-delusional nature of BIID, such that these individuals *know* what their legs look like, they *know* that they are part of the body, and they *know* that the desire to structurally or functionally abolish them is bizarre, but nevertheless *feel* a longstanding wish to do so (28). This logic is borrowed from Romano et al. (28) who hypothesized (and subsequently found) that although people with amputation-variant BIID could judge the limb as part of oneself, they had a reduced skin conductance response to painful stimuli approaching the unwanted limb (reflecting its internal underrepresentation). Our results, at least partly, confirm their hypothesis insofar that people with BIID can distinguish between the judgment of and (on the contrary) the feeling of what constitutes the boundaries of their bodily selves. Our results might also suggest that BIID is not a (psychiatric) body image disorder, per se, like anorexia nervosa, bulimia nervosa, and body dysmorphic disorder show distortions in making conscious judgments about body size and shape (42, 61). Noteworthy though is that in these disorders, body size or appearance is the critical factor that drives the underlying desire to modify the body. People with BIID do not feel that their leg is too big or that there is something wrong with the appearance of it. However, they do report that they feel “overcomplete” in the current body. This overcomplete feeling is not reflected in the conscious visual perception of the legs, however. Our data demonstrates that BIID individuals have a normal visual percept of their legs, thereby suggesting that the wish to amputate or paralysis the limb is not due to a distorted visual body image. Noteworthy, however, is that we did not include a control body part (e.g. hand) for those who desired paralysis or bilateral amputation. Given that the data for both the affected and unaffected limbs of the unilateral-desire participants fell within range of controls, we have reason to believe that it would be similar for the unaffected body parts of others. Nevertheless, these results should be considered with caution and might best serve simply as a starting point for understanding visual body perception in BIID.

Tactile perception of the affected (and unaffected) parts of the body was tested by asking participants to make judgments about the distance between two simultaneously applied tactile points on the legs, and for some participants, the arms. In this task, judging the distance between these points not only relies on primary tactile input (i.e. contact with the skin), but the reference to and communication with a mental model of the leg itself (37, 62, 63). In the tactile estimation task, participants must first 1) detect the two stimuli, and 2) refer to an internal (higher-order) model of the body that houses the model of the limb and 3) shape the hands to reflect the perceived distance. One study showed that participants with amputation-variant BIID have reduced PMC activity to tactile stimuli on the affected, but not the unaffected, leg (20). Moreover, another study showed decreased SPL activity when tactile stimuli was applied to the legs (regardless if it was the affected or unaffected leg) in individuals with BIID compared to controls (10). These results suggest that there might be issues with integrating primary tactile input on the lower body into a coherent body representation in BIID. In line with this, Aoyama and colleagues (5) showed that individuals with amputation-variant BIID show exaggerated attention to tactile stimuli on the affected part – that is, for two vibro-tactile taps to be perceived as simultaneous on the legs, the vibration had to be applied to the unaffected part first. Again, these findings might speak to an altered higher-order tactile representation of the limb. So, how might this affect tactile distance estimates on the affected body part? Previous studies have used the tactile estimation task to test this higher-order tactile representation in individuals with a disrupted or unhealthy bodily experience (39, 40, 64). Individuals with Anorexia Nervosa, for example, overestimate distances between two unseen tactile points on body, reflecting an internal oversized model of the body (39, 40). If the internal model of the leg in higher-order representations is missing in BIID, then distance estimates might rely on an earlier stage of processing touch, like in SI. In SI, cortical area devoted to the leg is much smaller than for more sensitive body parts, like the hand (65, 66). These “homuncular” distortions are corrected for, to some extent, by a visual representation of the body (i.e. tactile-visual remapping), such that the size of the body part, if properly represented in a higher-level stage of processing [(presumably posterior parietal areas), should reduce the distortion (for review see 67)]. The leg is physically much larger, though less sensitive, than the hand, and so one would assume that these more “proportional” maps of the legs in the higher-order areas could counteract this. In BIID, we might expect that if distances are judged without this correction factor, they might be perceived as much smaller than we would see in controls. However, we found that tactile distance perception for BIID participants was within range of normal controls. As discussed above, visual representations of the body in BIID participants were unimpaired compared to controls, which might account for the null results here. Our findings regarding directional distortions (for the arm and shin) are also in line with previous reports – such that stimuli applied in the vertical direction are underestimated more than those applied in the horizontal direction (33, 53, 54, 68). This mimics the underlying geometry of the, presumably oval-shaped, tactile receptive fields on the hairy skin (68–70). Therefore, these findings also reiterate that the impairment in BIID is not at the primary somatosensory level [in line with previous studies, (5)], as all participants were able to detect the tactile stimuli on the body.

In fact, our findings regarding somatosensory-related aspects of body representation complement the existing literature on BIID. Other studies have already shown that BIID is not likely caused by an underlying deficit related to the somatosensory representation. For example, symptoms of BIID are not diminished after caloric vestibular stimulation (CVS). CVS is a technique where cold water is irrigated through the ear canal in an attempt to modulate [higher-order somatosensory) representations of the body, e.g. (71–73)]. CVS has proven useful in correcting other aberrant experiences of the bodily self, such as in somatoparaphrenia [e.g. (74)], where a body part is experienced as foreign following stroke in the right hemisphere (75). Furthermore, people with BIID show exaggerated physiological responses to painful stimuli contacting the unwanted limb (9, 28), and they have a more vivid rubber foot illusion for the unwanted foot (8). In other words: stimulating the vestibular system does not change the symptoms, touch is still processed (albeit in a more exaggerated way) on the affected limb, and they can still temporarily experience a rubber foot as their own via multisensory (visuo-tactile) integration. While these somatosensory-related representations may be altered at a behavioral (5, 8) and a neural (10, 20) level, they do not seem to be deficient. Our data further exemplifies this.

Body representations underlying proprioceptive input and imagery were tested by asking participants to localize unseen landmarks on their legs. As the lower part of the legs (i.e. knee to shin) were affected in all BIID participants, we compared the perceived shape of this part in BIID participants to controls. Control participants judged the shins to be shorter than they are in reality, consistent with our previous report (33), and also consistent with judgments of tactile distance on this part of the body. We expected BIID participants to exhibit an even shorter/squatter perception, as this part of the body seems to be “missing” in the internal image of the self in BIID. We found no differences between BIID participants and controls on this task. However, in this case, participants could rely on sensory feedback (proprioceptive input) to judge the location of these points on the leg. Sensory feedback is overall normal in individuals with BIID, therefore it is plausible that this information compensates and/or overrides the internal disturbed model of the legs to facilitate judgments about the locations, similar to what we might have observed in tactile distance estimates. Thus, we also included a condition where participants were asked to imagine their legs outstretched under the display screen and to judge where these landmarks would be (33, 55). Therefore, instead of integrating proprioceptive input with a stored model of the body, one must therefore use proprioceptive imagery to localize these landmarks. In such a case, participants have to rely on an imagined model of the limb (55). Localizing landmarks on the imagined leg yielded similar distortions in BIID participants as controls (and also similar to the Real condition, when the leg was under the screen). In line with this, we recently showed that individuals with BIID perform similarly to intact controls and lower-limb amputees during mental rotation of feet (Stone et al. (76), *preprint*), a task which involves mentally rotating your limb to match the posture of the pictured limb in order to make a judgment. Therefore, the findings of the current study support a preserved ability to imagine the lower limbs in a different posture than the current one in individuals with BIID. One participant (2-RA, right leg amputation desire) did elicit extremely high NSI scores on the Imagine condition for both legs. Inspection of the data revealed that this was a product of localizing the knee and ankle in (nearly) the same location, almost as though the shape mimicked that of an amputated/underdeveloped lower leg. However, as we did not see this in all of the BIID participants, and it was not specific to his (right) to-be-removed leg, we do not suspect that this was reflective of BIID, per se. Noteworthy is that this participant had PDD-NOS, a form of Autism Spectrum Disorder (77) in addition to BIID, which might have influenced imagery of one’s own body position (78–80). It is also possible, unfortunately, that the participant simply misunderstood the Imagine condition instructions. Finally, participants were also asked to localize landmarks on an unseen corporeal object (mannequin leg). Judgments about the fake leg did not differ between BIID participants and controls. Thus, while individuals with BIID might report having an incongruent physical and mental body representation, it does not seem to interact with the implicit perception of the configuration of the leg, whether it be their own legs, or a foreign leg.

It is worth noting that the overall perceived configurations of the lower legs in all conditions were indeed distorted, insofar that distances between estimated landmarks in the length direction, compared to the actual distance between the landmarks, were underestimated more than the (average of) those in the width direction (made evident by the NSIs > 1, representing a leg that is wider than it is long). This replicates our previous study (33) and is also in line with how individuals perceive the internal configuration of their hands (29, 34, 51, 67, 81–84). Our current findings therefore suggest that leg representations in BIID, albeit distorted with respect to their physical size, are overall “normal” in this population. This could be informative for clinicians, insofar that tests of leg perception in BIID should yield normal results. If not, then another factor might be at play in the wish to amputate or paralyze the limb.

The tasks employed in the current study were mainly perceptual in nature. Specifically, they involved making judgments about the properties of the body, providing us with a description of the leg representation. There is an ongoing discussion regarding the dissociation between types of body representations (37, 85–89). Longo (36, 38) has proposed that there are not necessarily clear distinctions between body representations, but rather that they exist along a continuum, with implicit (e.g. distorted body model underlying tactile/proprioceptive information) and explicit (visually-based veridical) ones on opposite ends of the continuum. It is thus the “different weightings” of these models of the body that underlie body representations on the continuum [page 386, (36)]. People with BIID desire non-action (and consequently no somatosensory/proprioceptive feedback) of the affected part, either by completing removing the limb or paralyzing it. It could be that body representations that tap more into the function of the limb (perhaps somewhere in the middle of Longo’s proposed continuum), rather than the perception, would better capture the breakdown (if there is one) of body representation underlying BIID.

These action- and perception-related aspects of body representations might be better considered under the (somatosensory) dorsal-ventral stream model proposed by Dijkerman and de Haan (85). They suggested that somatosensory processes have functional and anatomical “what/ventral” and “how/dorsal” pathways (similar to that in vision). That is, the dorsal pathway processes aspects of the body representation for the guidance of action, while the ventral pathway is related to perception and memory about the body. Our tasks involved making spatial localization/metric perceptual judgments about the body (e.g. localization and tactile estimation tasks) using responses (e.g. index finger-thumb separation) that were similar to those used to assess ventral stream functions in the visual domain (90). In this way, results were probably reflecting the (perceptual) ventral representation. Tasks that tap chiefly into dorsal stream functions, whether it be underlying somatosensory (85) or visual (91) input about the limbs might be more informative in BIID. For example, tasks involving motor control of the legs, such as locomotion (92, 93), obstacle avoidance (94), object interaction (e.g. football), proprioceptive matching (95), postural manipulations, or kinematics of goal-directed “reach-to-touch” movements (i.e. because the lower limbs do not “grasp”). However, since many people with BIID spend a lot of time pretending to be in the body they desire [i.e. by binding up the leg to simulate an amputation, or using a wheelchair (1, 6, 96)], they use their leg(s) much less, which might in and of itself affect outcomes on body representation tasks related to body action [e.g. (97, 98)]. Also noteworthy is that immobilizing the limb affects plasticity of sensorimotor brain areas (99), so it is possible that some of the body representation network disturbances revealed in neuroimaging studies of BIID participants could be partly due to long-term pretending behaviors (11), i.e. a product of behavior rather than a cause of BIID. Therefore, future studies should explore action-related leg representations in BIID, while carefully considering pretending behaviors too.

Finally, this is the first study, to our knowledge, that has tested amputation- and paralysis-variant BIID participants involving behavioral measurements. All of the behavioral studies on BIID, to date, have been in those who desire amputation (5, 7, 8, 100). A few questionnaire-based investigations of BIID have included paralysis-variant participants (4, 6, 101) and only one neuroimaging study included paralysis-variant participants (*n* = 2) in addition to amputation-desire participants (*n* = 6; 12). The questionnaire data suggested that the only major difference, besides the desired body type, between amputation- and paralysis-variant BIID individuals seems to be a higher prevalence of women who desire paralysis versus amputation. One of our paralysis-variant participants was indeed female, but we had another who was biologically male but desired to be female in addition to desiring paraplegia. The parallels between Gender Dysphoria and BIID have been discussed elsewhere (14, 96). Whether the desire to paralyze the legs is intertwined with the desire to modify the gender could probably only be confirmed after surgical intervention addressing one of the two conditions. Neuroimaging data including paralysis-variant BIID participants showed structural alterations in the premotor cortex and posterior cerebellum, compared to controls, but the data could not be analyzed separately for each variant of BIID because there were only 2 paralysis-variant participants included. While we also could not conduct a group-level analysis comparing the two variants (due to sample size limitations), our single-case analysis did not suggest that the paralysis- and amputation-variants differed in task performance. Specifically, our findings suggest that the amputation- and paralysis-variants are similar in their perception of the affected parts of their body, so if a dissociation between the two variants exists, it does not lie in how one perceives the size/shape of the legs. However, larger-scale studies comparing (and correlating task performance with) the two variants might provide a more thorough understanding of the mechanisms that underlie BIID.

In conclusion, body perception is a combination of central, presumably stable, representations of the body and peripheral input, which are more transient in nature (102). These results suggest that the peripheral input, seemingly superfluous for people with BIID, preserves perceptions of the body that are like the general population. Our data cannot confirm or deny the presence of an atrophic representation of the affected body part(s) in BIID but does suggest that lower limb perception is not disturbed in this population. The finding that the perception of the leg size/shape is normal might align with the fact that the limb itself is healthy and functions normally. Thus while people with BIID feel that part of the body is foreign, they can still make normal sensory-guided implicit and explicit judgments about the limb, two components of the bodily experience that may be dissociable in nature [e.g. (103)].

## Data Availability Statement

The datasets generated for this study are available on request to the corresponding author.

## Ethics Statement

This study was carried out in accordance with the recommendations of ‘name of guidelines, name of committee’ with written informed consent from all subjects. All subjects gave written informed consent in accordance with the Declaration of Helsinki. The protocol (number: FETC 17-004) was approved by Utrecht University’s local ethics committee.

## Author Contributions

KS contributed to experimental design, data collection, data analysis, and writing/editing of manuscript. KS wrote the manuscript with input from all authors. CK and ME assisted with data collection and editing of manuscript. RB assisted with participant recruitment, provided feedback on the experimental design, and edited the manuscript. AK and HD contributed to experimental design, writing, and editing the manuscript. AK and HD also supervised the project. All authors read and approved of the final manuscript.

## Funding

This study was supported by a Netherlands Organisation for Scientific Research (NWO) talent grant (406.15.282) and a Natural Sciences and Engineering Research Council of Canada (NSERC) Postgraduate Scholarship.

## Conflict of Interest

The authors declare that the research was conducted in the absence of any commercial or financial relationships that could be construed as a potential conflict of interest.

## Appendix 1

### BIID Screening – Criteria

Must meet the following criteria (1):

- An intense and persistent desire to become physically disabled in a significant way (e.g., major limb amputee, paraplegic, blind), with onset by early adolescence
- Persistent discomfort or intense feelings of inappropriateness concerning current nondisabled body configuration
- The desire to become physically disabled results in harmful consequences, as manifested by either (or both) of the following:
  1. The preoccupation with the desire (including time spent pretending to be disabled) significantly interferes with productivity, with leisure activities, or with social functioning (e.g., person is unwilling to have a close relationship because it would make it difficult to pretend)
  2. Attempts to actually become disabled have resulted in the person putting his or her health or life in significant jeopardy
- The desire to become disabled is not primarily motivated by sexual arousal or by any perceived advantages of becoming disabled
- The disturbance is not a manifestation of a psychotic process (e.g., desire to amputate a limb because of delusional conviction that the limb belongs to another person), is not due to a primary neurological condition such as post stroke neglect syndrome and is not better accounted for by another mental disorder such as Body Dysmorphic Disorder or Factitious Disorder

Subtype based on predominant desired disability

Amputation type
Paraplegia type
Other type
Unspecified type

Other:

- Normal tactile sensitivity (e.g. no excessive scarring on affected body part(s) which might influence tactile sensitivity)
- No body image disorder (i.e. no eating disorders or BDD)

Further questions:

1. At what age did your BIID start?
2. Can you describe your BIID?
3. What is the primary reason you want to change your body?
4. Does your BIID interfere with your everyday life? If so, can you describe in what ways?
5. Have you ever visited a psychiatrist?
6. Have you been diagnosed with a psychiatric or somatic disorder?
7. Are you currently using any medications?
8. Do you currently use drugs or alcohol?
9. Have you ever had a head injury? If so, can you describe when and how it happened?
10. Do you have any scars on the affected body part(s)? If so, can you describe their locations exactly?
11. Do you have trouble detecting touch sensations on any part(s) of your body? If so, where exactly?
12. Do you have normal vision? If not, has your vision been corrected with lenses, glasses, surgery, etc.?

References

1 First MB, Fisher CE. Body Integrity Identity Disorder: The persistent desire to acquire a physical disability. Psychopathology (2012) 45:3–14. doi:10.1159/000330503
