# Supplementary material for: An Investigation of Lower Limb Representations Underlying Vision, Touch, and Proprioception in Body Integrity Identity Disorder
Source: Front Psychiatry. 2020 Feb 25;11:15. doi: 10.3389/fpsyt.2020.00015 (PMC7052367; doi:10.3389/fpsyt.2020.00015)
Supplement: Supplementary file 1 [file DataSheet_1.docx]

***Supplementary Material: Stone et al., 2020***

**Supplementary figures**

**Tactile distance estimates**

The following figures display the estimates (in mm) per distance applied for the shin and arms. The bars have been overlaid with individual points of the control group (figures S1, S3) and the BIID participants (figures S2, S4). Participants, in general, opened their hands wider for larger distances.


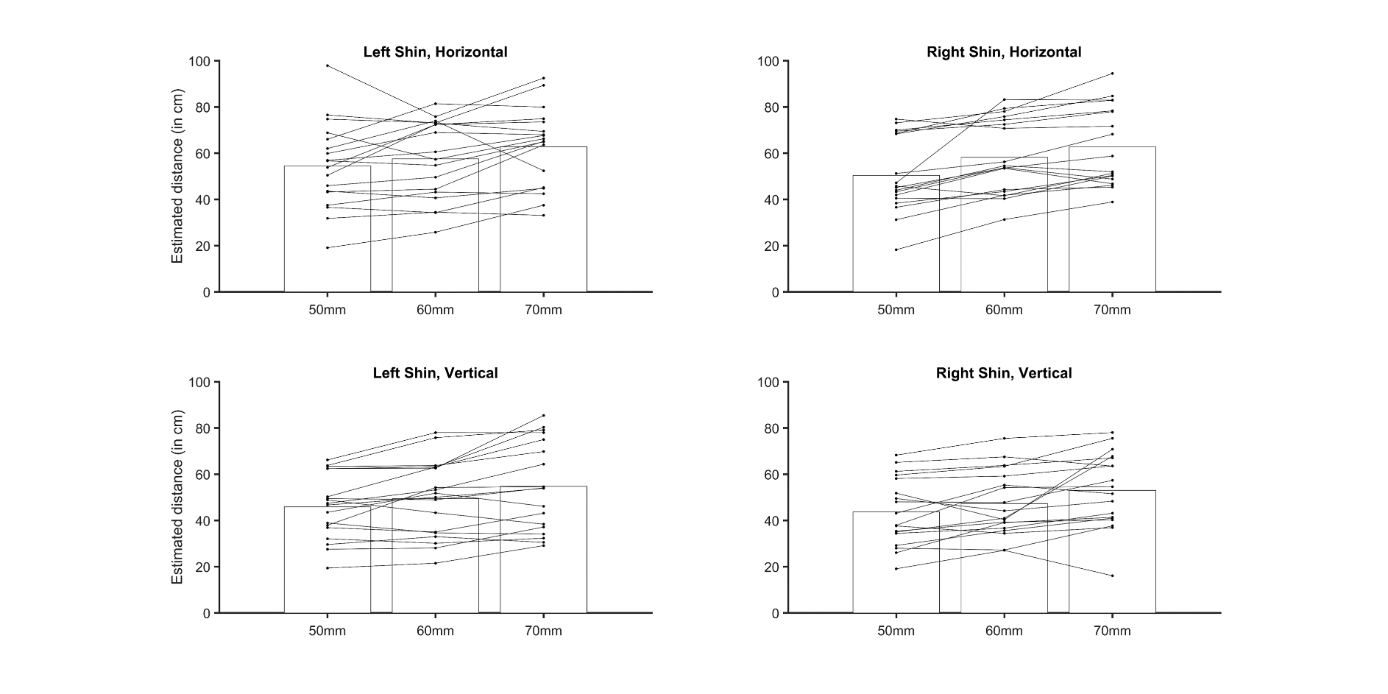


**Figure S1.** Bar groups displaying average estimated distances for each applied distance to the shin in the horizontal (top panel) and vertical (bottom panel) directions. Individual data points for each control participant is plotted on top to show pattern of estimates.


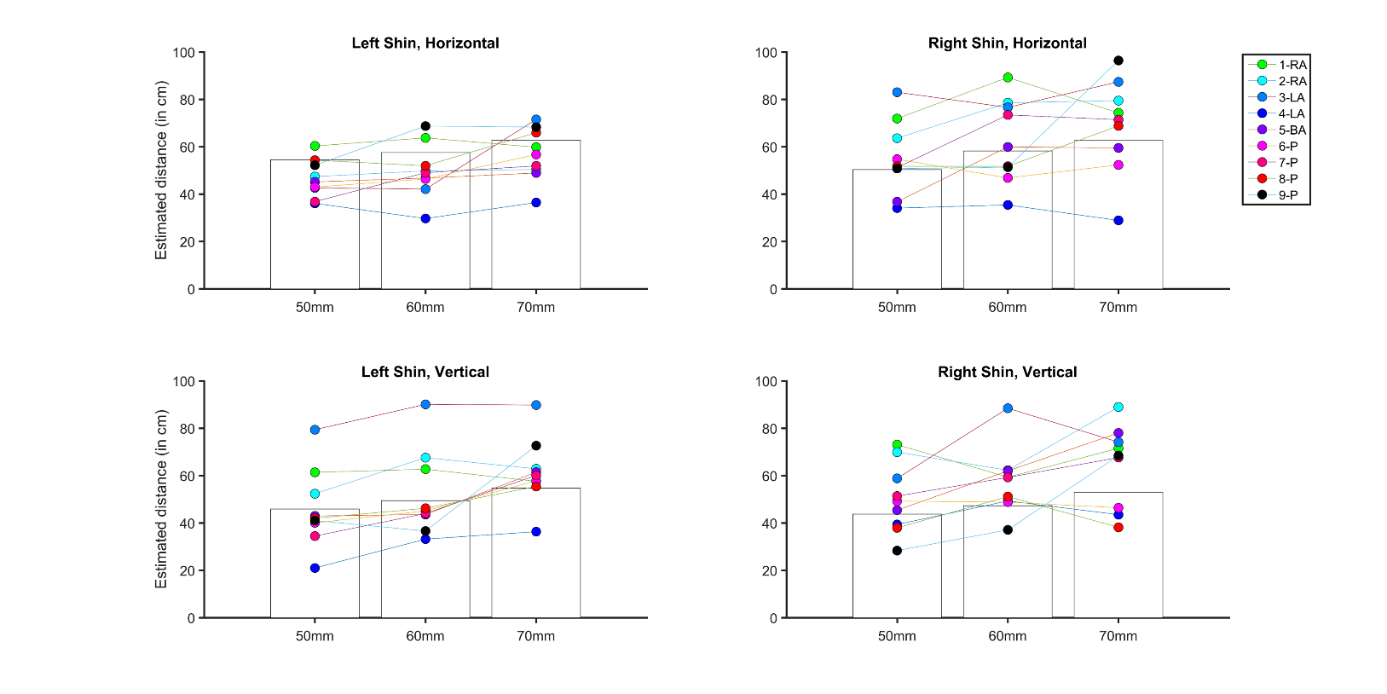
**Figure S2.** Bar groups displaying average estimated distances for each applied distance to the shin in the horizontal (top panel) and vertical (bottom panel) directions for the control group. Individual data points for each BIID participant is plotted on top to show pattern of estimates. The legend on the top right indicates each participant code and colour. RA = right amputation desire; LA = left amputation desire; BA = bilateral amputation desire; P = paralysis desire.


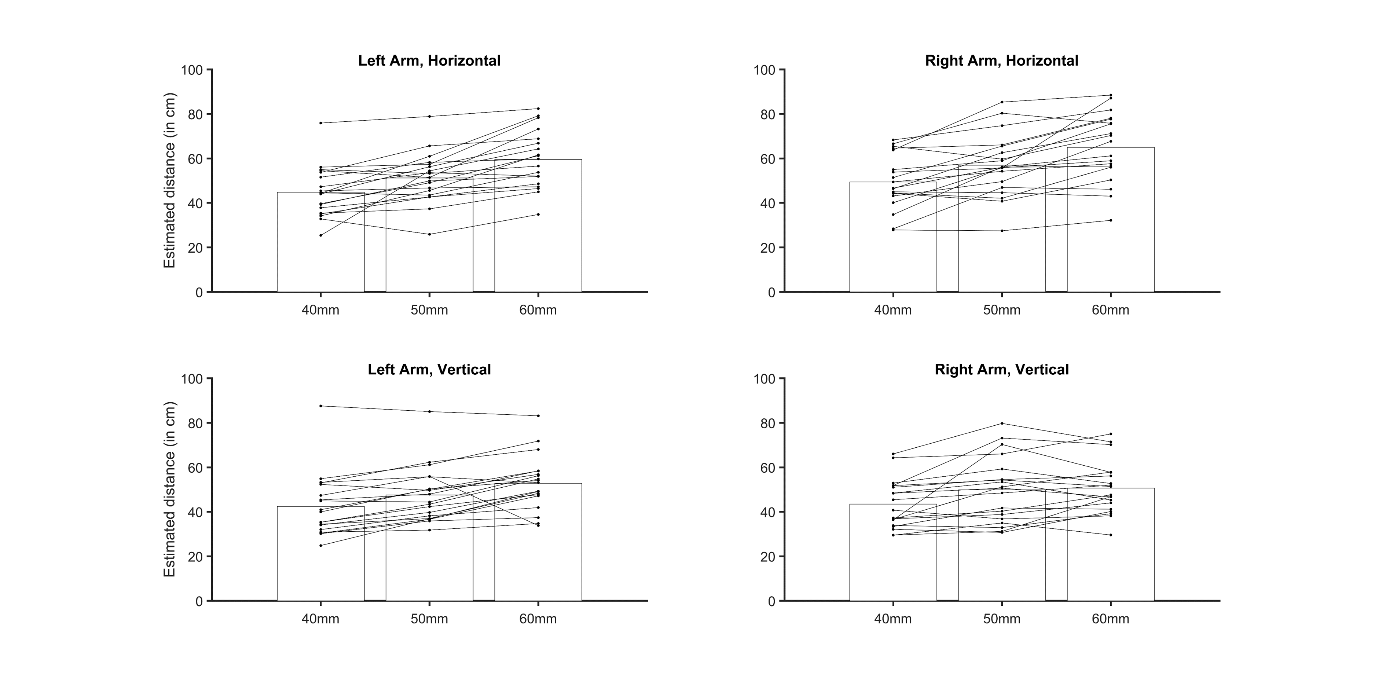


**Figure S3.** Bar groups displaying average estimated distances for each applied distance to the forearm in the horizontal (top panel) and vertical (bottom panel) directions. Individual data points for each control participant is plotted on top to show pattern of estimates.


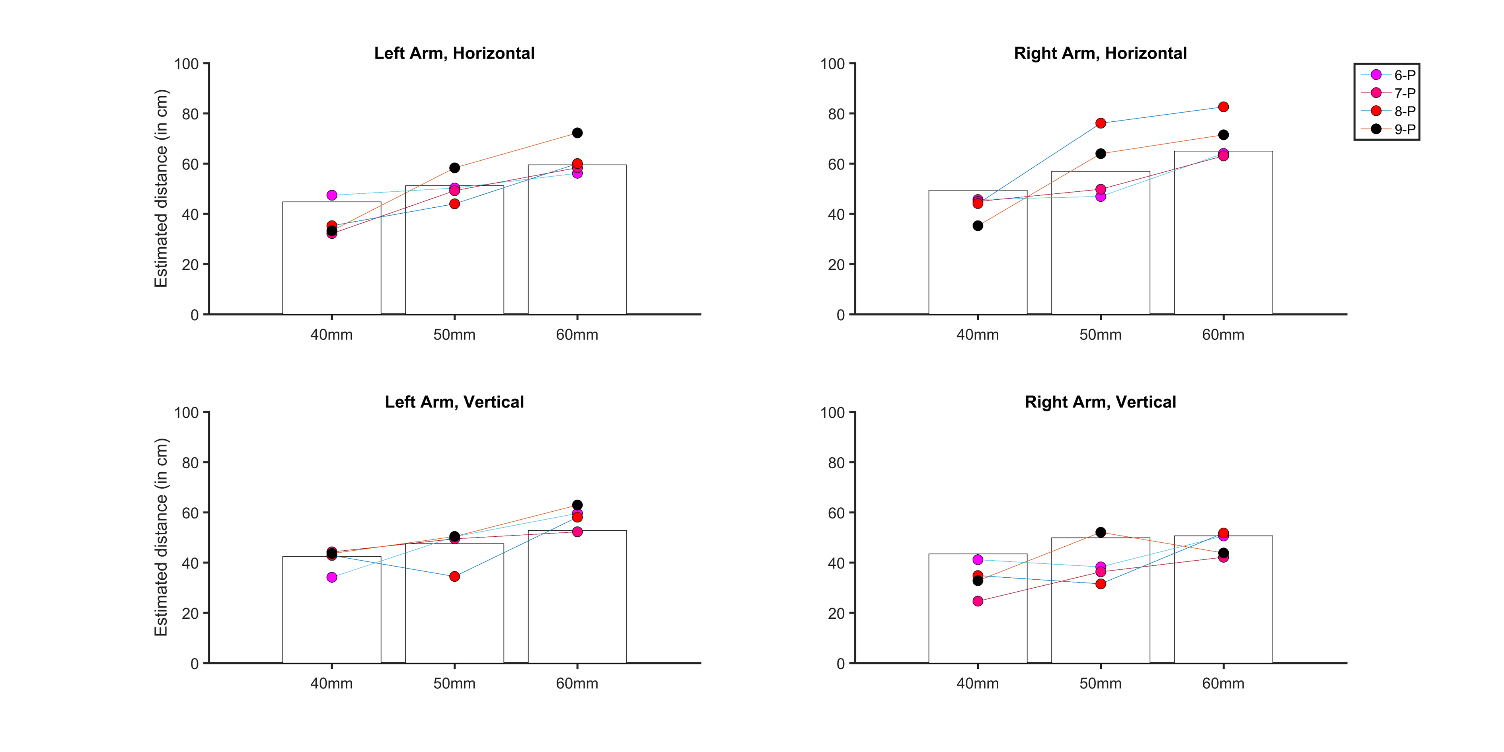


**Figure S4.** Bar groups displaying average estimated distances for each applied distance to the forearm in the horizontal (top panel) and vertical (bottom panel) directions for the control group. Individual data points for each paralysis-desire BIID participant is plotted on top to show pattern of estimates. The legend on the top right indicates each participant code and colour. P = paralysis desire.

**Supplementary tables**

The following are tables with the p-values, credible intervals, and effect sizes for comparing each BIID participant to the control sample using Crawford-Garthwaite Bayesian single-case t-tests. Significant results are marked with an asterisk (*).

1. **Template Matching Task**

Single-case comparison between average scores of controls and individual BIID participant scores.

We hypothesized that BIID participants would overestimate their affected legs more than controls. Therefore, where *p* < 0.05, but effect size is negative, it suggests that the BIID participant scored lower than controls (but due to one-sided testing, this is not relevant for the current analysis).

**Table S1.1.** Condition: right leg width

| Participant | *p-value* | lower CI | upper CI | effect size (z) |
| --- | --- | --- | --- | --- |
| 1-RA | 0.14 | 0.04 | 0.26 | -1.15 |
| 2-RA | 0.39 | 0.26 | 0.50 | 0.26 |
| 3-LA | 0.27 | 0.13 | 0.42 | 0.63 |
| 4-LA | 0.10 | 0.02 | 0.21 | -1.33 |
| 5-BA | 0.30 | 0.16 | 0.46 | 0.54 |
| 6-P | 0.43 | 0.33 | 0.50 | 0.03 |
| 7-P | 0.24 | 0.10 | 0.40 | 0.73 |
| 8-P | 0.25 | 0.10 | 0.40 | -0.70 |
| 9-P | 0.34 | 0.22 | 0.50 | -0.41 |

**Table S1.2.** Condition: right leg length

| Participant | *p-value* | lower CI | upper CI | effect size (z) |
| --- | --- | --- | --- | --- |
| 1-RA | 0.35 | 0.22 | 0.50 | -0.39 |
| 2-RA | 0.22 | 0.09 | 0.36 | -0.82 |
| 3-LA | 0.04 | 0.00 | 0.10 | -1.93 |
| 4-LA | 0.12 | 0.03 | 0.23 | 1.23 |
| 5-BA | 0.20 | 0.07 | 0.33 | -0.89 |
| 6-P | 0.38 | 0.25 | 0.50 | -0.30 |
| 7-P | 0.42 | 0.31 | 0.50 | 0.14 |
| 8-P | 0.07 | 0.00 | 0.15 | 1.60 |
| 9-P | 0.41 | 0.29 | 0.50 | 0.20 |

**Table S1.3.** Condition: left leg width

| Participant | *p-value* | lower CI | upper CI | effect size (z) |
| --- | --- | --- | --- | --- |
| 1-RA | 0.18 | 0.07 | 0.32 | -0.94 |
| 2-RA | 0.09 | 0.01 | 0.19 | 1.41 |
| 3-LA | 0.20 | 0.07 | 0.34 | 0.89 |
| 4-LA | 0.01 | 0.00 | 0.05 | -2.41 |
| 5-BA | 0.42 | 0.31 | 0.50 | -0.15 |
| 6-P | 0.12 | 0.03 | 0.24 | -1.21 |
| 7-P | 0.14 | 0.04 | 0.26 | 1.12 |
| 8-P | 0.17 | 0.05 | 0.31 | -0.99 |
| 9-P | 0.38 | 0.25 | 0.50 | -0.30 |

**Table S1.4.** Condition: left leg length

| Participant | *p-value* | lower CI | upper CI | effect size (z) |
| --- | --- | --- | --- | --- |
| 1-RA | 0.42 | 0.31 | 0.50 | -0.13 |
| 2-RA | 0.29 | 0.14 | 0.44 | -0.59 |
| 3-LA | 0.10 | 0.01 | 0.19 | -1.39 |
| 4-LA | 0.10 | 0.02 | 0.20 | 1.35 |
| 5-BA | 0.31 | 0.16 | 0.47 | -0.52 |
| 6-P | 0.28 | 0.14 | 0.44 | 0.60 |
| 7-P | 0.35 | 0.23 | 0.50 | -0.39 |
| 8-P | 0.19 | 0.07 | 0.32 | 0.94 |
| 9-P | 0.34 | 0.21 | 0.49 | 0.41 |

1. **Tactile Estimation Task**

Single-case comparison between average percent misestimations of controls and individual BIID participant scores.

We hypothesized that BIID participants would underestimate distances on their affected legs more than controls. Therefore, where *p* < 0.05, but effect size is positive, it suggests that the BIID participant scored higher than controls (but due to one-sided testing, this is not relevant for the current analysis).

**Table S2.1.** Condition: Right shin, horizontal

| Participant | *p-value* | lower CI | upper CI | effect size (z) |
| --- | --- | --- | --- | --- |
| 1-RA | 0.09 | 0.01 | 0.20 | 1.40 |
| 2-RA | 0.15 | 0.04 | 0.28 | 1.10 |
| 3-LA | 0.06 | 0.00 | 0.14 | 1.65 |
| 4-LA | 0.07 | 0.01 | 0.16 | -1.58 |
| 5-BA | 0.37 | 0.24 | 0.50 | -0.32 |
| 6-P | 0.36 | 0.23 | 0.50 | -0.37 |
| 7-P | 0.30 | 0.16 | 0.46 | 0.55 |
| 8-P | 0.43 | 0.33 | 0.50 | 0.03 |
| 9-P | 0.28 | 0.13 | 0.44 | 0.60 |

**Table S2.2.** Condition: Left shin, horizontal

| Participant | *p-value* | lower CI | upper CI | effect size (z) |
| --- | --- | --- | --- | --- |
| 1-RA | 0.39 | 0.26 | 0.50 | 0.26 |
| 2-RA | 0.32 | 0.18 | 0.49 | -0.48 |
| 3-LA | 0.38 | 0.25 | 0.50 | -0.30 |
| 4-LA | 0.09 | 0.01 | 0.19 | -1.41 |
| 5-BA | 0.28 | 0.13 | 0.44 | -0.62 |
| 6-P | 0.31 | 0.16 | 0.47 | -0.51 |
| 7-P | 0.26 | 0.11 | 0.41 | -0.68 |
| 8-P | 0.43 | 0.33 | 0.50 | 0.02 |
| 9-P | 0.35 | 0.23 | 0.50 | 0.37 |

**Table S2.3.** Condition: Right shin, vertical

| Participant | *p-value* | lower CI | upper CI | effect size (z) |
| --- | --- | --- | --- | --- |
| 1-RA | 0.08 | 0.01 | 0.17 | 1.50 |
| 2-RA | 0.04 | 0.00 | 0.10 | 1.93 |
| 3-LA | 0.04 | 0.00 | 0.10 | 1.93 |
| 4-LA | 0.39 | 0.26 | 0.50 | -0.27 |
| 5-BA | 0.16 | 0.04 | 0.29 | 1.05 |
| 6-P | 0.43 | 0.33 | 0.50 | 0.04 |
| 7-P | 0.20 | 0.08 | 0.35 | 0.87 |
| 8-P | 0.35 | 0.22 | 0.50 | -0.39 |
| 9-P | 0.40 | 0.28 | 0.50 | -0.22 |

**Table S2.4.** Condition: Left shin, vertical

| Participant | *p-value* | lower CI | upper CI | effect size (z) |
| --- | --- | --- | --- | --- |
| 1-RA | 0.24 | 0.10 | 0.39 | 0.74 |
| 2-RA | 0.23 | 0.10 | 0.38 | 0.76 |
| 3-LA | 0.02 | 0.00 | 0.05 | 2.39 |
| 4-LA | 0.13 | 0.03 | 0.25 | -1.20 |
| 5-BA | 0.43 | 0.33 | 0.50 | 0.02 |
| 6-P | 0.43 | 0.32 | 0.50 | -0.08 |
| 7-P | 0.41 | 0.29 | 0.50 | -0.18 |
| 8-P | 0.43 | 0.32 | 0.50 | -0.07 |
| 9-P | 0.43 | 0.32 | 0.50 | 0.07 |

**Table S2.5.** Condition: Right arm, horizontal

| Participant | *p-value* | lower CI | upper CI | effect size (z) |
| --- | --- | --- | --- | --- |
| 6-P | 0.35 | 0.22 | 0.50 | -0.38 |
| 7-P | 0.36 | 0.23 | 0.50 | -0.35 |
| 8-P | 0.22 | 0.09 | 0.37 | 0.80 |
| 9-P | 0.43 | 0.32 | 0.50 | -0.02 |

**Table S2.6.** Condition: Left arm, horizontal

| Participant | *p-value* | lower CI | upper CI | effect size (z) |
| --- | --- | --- | --- | --- |
| 6-P | 0.43 | 0.32 | 0.50 | -0.06 |
| 7-P | 0.32 | 0.17 | 0.48 | -0.49 |
| 8-P | 0.31 | 0.16 | 0.47 | -0.51 |
| 9-P | 0.39 | 0.26 | 0.50 | 0.25 |

**Table S2.7.** Condition: Right arm, vertical

| Participant | *p-value* | lower CI | upper CI | effect size (z) |
| --- | --- | --- | --- | --- |
| 6-P | 0.43 | 0.33 | 0.50 | 0.00 |
| 7-P | 0.43 | 0.32 | 0.50 | 0.05 |
| 8-P | 0.39 | 0.26 | 0.50 | -0.24 |
| 9-P | 0.36 | 0.23 | 0.50 | 0.36 |

**Table S2.8.** Condition: Left arm, vertical

| Participant | *p-value* | lower CI | upper CI | effect size (z) |
| --- | --- | --- | --- | --- |
| 6-P | 0.36 | 0.23 | 0.50 | -0.35 |
| 7-P | 0.16 | 0.04 | 0.28 | -1.07 |
| 8-P | 0.26 | 0.12 | 0.43 | -0.66 |
| 9-P | 0.35 | 0.22 | 0.50 | -0.38 |

*Condition: right and left thighs (above demarcation) for participant 5-BA*

**Table S2.9.** Right thigh, horizontal

| Participant | *p-value* | lower CI | upper CI | effect size (z) |
| --- | --- | --- | --- | --- |
| 5-BA | 0.14 | 0.03 | 0.28 | 1.1 |

**Table S2.10.** Right thigh, vertical

| Participant | *p-value* | lower CI | upper CI | effect size (z) |
| --- | --- | --- | --- | --- |
| 5-BA | 0.30 | 0.14 | 0.46 | -0.53 |

**Table S2.11.** Left thigh, horizontal

| Participant | *p-value* | lower CI | upper CI | effect size (z) |
| --- | --- | --- | --- | --- |
| 5-BA | 0.39 | 0.27 | 0.49 | -0.21 |

**Table S2.12.** Left thigh, vertical

| Participant | *p-value* | lower CI | upper CI | effect size (z) |
| --- | --- | --- | --- | --- |
| 5-BA | 0.23 | 0.09 | 0.39 | 0.74 |

1. **Localization Task**

Single-case comparison between average lower leg normalized shape index scores of controls and individual BIID participant scores.

We hypothesized that BIID participants would have higher NSIs (i.e. underestimate the length with respect to width) for their affected legs more than controls.

**Table S3.1.** Condition: Right leg, Real

| Participant | *p-value* | upper-CI | lower-CI | effect size (z) |
| --- | --- | --- | --- | --- |
| 1-RA | 0.11 | 0.02 | 0.23 | 1.29 |
| 2-RA | 0.38 | 0.25 | 0.5 | 0.29 |
| 3-LA | 0.29 | 0.13 | 0.44 | -0.58 |
| 4-LA | 0.42 | 0.31 | 0.5 | -0.11 |
| 5-BA | 0.38 | 0.24 | 0.5 | 0.31 |
| 6-P | 0.42 | 0.31 | 0.5 | 0.11 |
| 7-P | 0.21 | 0.08 | 0.36 | -0.85 |
| 8-P | 0.21 | 0.08 | 0.36 | -0.84 |
| 9-P | 0.21 | 0.08 | 0.37 | -0.83 |

**Table S3.2.** Condition: Left leg, Real

| Participant | *p-value* | upper-CI | lower-CI | effect size (z) |
| --- | --- | --- | --- | --- |
| 1-RA | 0.33 | 0.2 | 0.5 | 0.45 |
| 2-RA | 0.4 | 0.27 | 0.5 | -0.23 |
| 3-LA | 0.43 | 0.33 | 0.5 | -0.01 |
| 4-LA | 0.42 | 0.3 | 0.5 | 0.13 |
| 5-BA | 0.31 | 0.17 | 0.48 | 0.51 |
| 6-P | 0.42 | 0.3 | 0.5 | -0.14 |
| 7-P | 0.16 | 0.04 | 0.3 | -1.05 |
| 8-P | 0.12 | 0.02 | 0.24 | -1.24 |
| 9-P | 0.22 | 0.09 | 0.37 | -0.82 |
|  |  |  |  |  |

**Table S3.3.** Condition: Right leg, Imagine

| Participant | *p-value* | upper-CI | lower-CI | effect size (z) |
| --- | --- | --- | --- | --- |
| 1-RA | <0.00001* | 0 | 0 | 127.9 |
| 2-RA | 0.26 | 0.12 | 0.42 | -0.67 |
| 3-LA | 0.43 | 0.32 | 0.5 | 0.04 |
| 4-LA | 0.34 | 0.21 | 0.5 | -0.43 |
| 5-BA | 0.15 | 0.04 | 0.29 | 1.08 |
| 6-P | 0.19 | 0.07 | 0.34 | -0.91 |
| 7-P | 0.34 | 0.21 | 0.5 | 0.41 |
| 8-P | 0.43 | 0.32 | 0.5 | 0.01 |
| 9-P | 0.35 | 0.22 | 0.5 | -0.38 |

**Table S3.4.** Condition: Left leg, Imagine

| Participant | *p-value* | upper-CI | lower-CI | effect size (z) |
| --- | --- | --- | --- | --- |
| 1-RA | <0.00001* | 0 | 0 | 25.43 |
| 2-RA | 0.23 | 0.09 | 0.38 | -0.8 |
| 3-LA | 0.2 | 0.06 | 0.34 | 0.88 |
| 4-LA | 0.42 | 0.31 | 0.5 | 0.1 |
| 5-BA | 0.26 | 0.11 | 0.42 | 0.67 |
| 6-P | 0.15 | 0.04 | 0.28 | -1.12 |
| 7-P | 0.22 | 0.08 | 0.37 | 0.81 |
| 8-P | 0.14 | 0.03 | 0.27 | -1.13 |
| 9-P | 0.34 | 0.21 | 0.5 | -0.43 |

**Table S3.5.** Condition: Mannequin

| Participant | *p-value* | upper-CI | lower-CI | effect size (z) |
| --- | --- | --- | --- | --- |
| 1-RA | 0.15 | 0.03 | 0.28 | 1.12 |
| 2-RA | 0.11 | 0.02 | 0.22 | -1.34 |
| 3-LA | 0.37 | 0.24 | 0.50 | 0.31 |
| 4-LA | 0.43 | 0.32 | 0.50 | -0.05 |
| 5-BA | 0.19 | 0.06 | 0.33 | -0.93 |
| 6-P | 0.34 | 0.21 | 0.50 | -0.43 |
| 7-P | 0.14 | 0.03 | 0.27 | -1.16 |
| 8-P | 0.14 | 0.04 | 0.27 | -1.13 |
| 9-P | 0.34 | 0.21 | 0.50 | -0.42 |
